# Supplementary material for: Evaluation of HLA Region-Specific High-Throughput Sequencing FASTQ Reads Combined with Ensemble HLA-Typing Tools for Rapid and High-Confidence HLA Typing
Source: Biology (Basel). 2025 Dec 1;14(12):1717. doi: 10.3390/biology14121717 (PMC12731231; doi:10.3390/biology14121717)
Supplement: Supplementary file 1 [file biology-14-01717-s001.zip › ReviewerManuscript-Version3-biology-3912409-Updated2-SupplementaryMaterial.pdf]

SUPPLEMENTARY FIGURES:

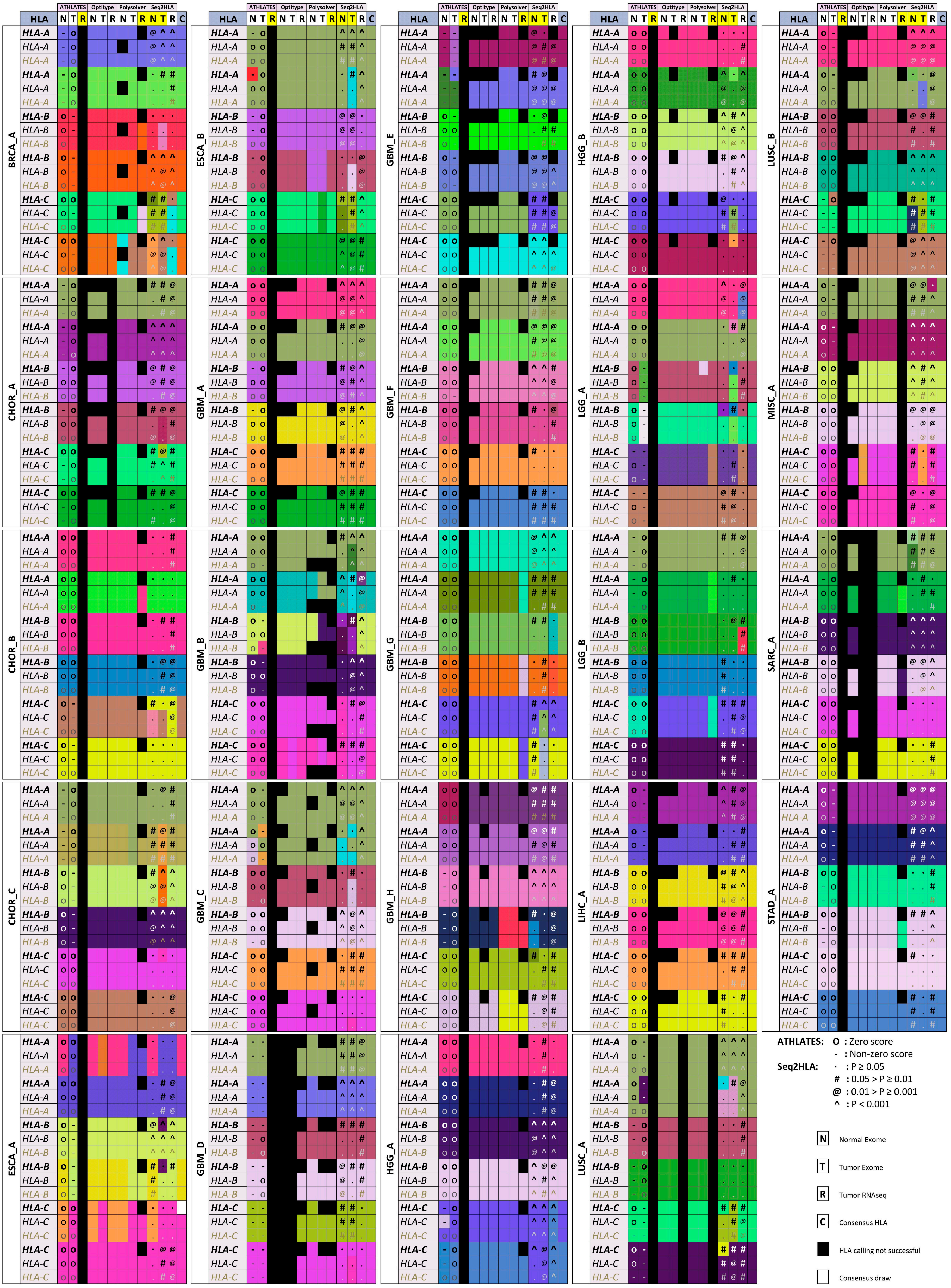

Supplementary Figure S1. HLA Class I HLA-typing results derived from using original FASTQ, All-HLA filtered FASTQ, and 9-HLA filtered FASTQ (Normal-DNA exome, Tumor-DNA exome and Tumor-RNA RNAseq) using four software tools for 24 patients. Each distinct two-field HLA call is masked with distinct color shade. Rows labeled in bold black, normal black and brown correspond to HLA-typing results from original FASTQ, All-HLA filtered FASTQ, and 9-HLA filtered FASTQ respectively.

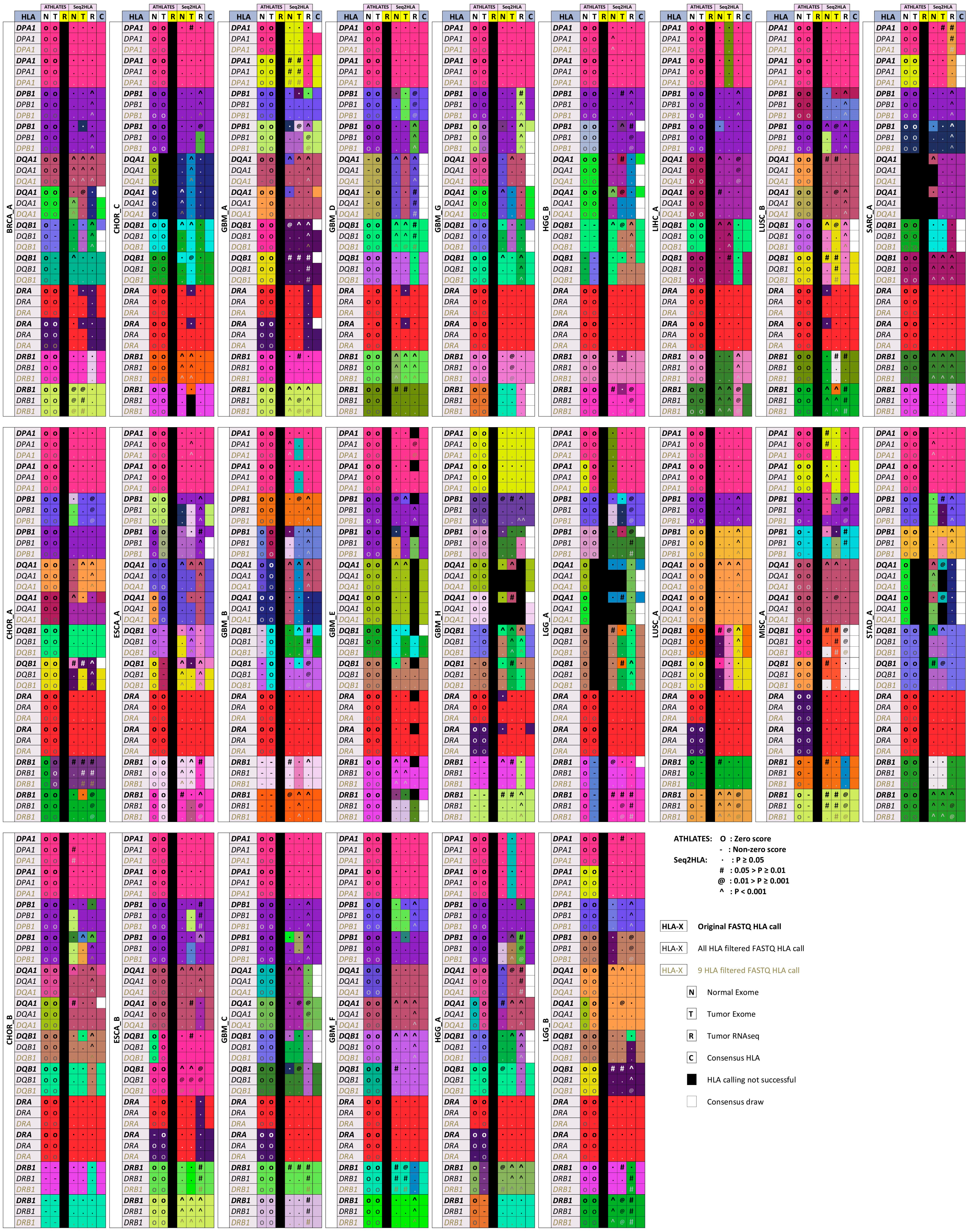

Supplementary Figure S2. HLA Class II HLA-typing results derived from using original FASTQ, All-HLA filtered FASTQ, and 9-HLA filtered FASTQ (Normal-DNA exome, Tumor-DNA exome and Tumor-RNA RNAseq) using two software tools for 24 patients. Each distinct two-field HLA call is masked with distinct color shade. Rows labeled in bold black, normal black and brown correspond to HLA-typing results from original FASTQ, All-HLA filtered FASTQ, and 9-HLA filtered FASTQ respectively.

A HLA Class I

Percent concordance

Scale: 20 | 40 | 60 | 80 | 90 | 95 | 100

Concordance

FASTQ	HLA	BRCA_A	CHOR_A	CHOR_B	CHOR_C	ESCA_A	ESCA_B	GBM_A	GBM_B	GBM_C	GBM_D	GBM_E	GBM_F	GBM_G	GBM_H	HGG_A	HGG_B	LGG_A	LGG_B	LIHC_A	LUSC_A	LUSC_B	MISC_A	SARC_A	STAD_A	Total %
**Original**	***HLA-A***																									
All-HLA	*HLA-A*	100.00	100.00	100.00	100.00	100.00	100.00	100.00	90.00	100.00	100.00	100.00	88.89	100.00	100.00	100.00	100.00	90.91	100.00	100.00	100.00	100.00	88.89	100.00	100.00	98.28
9-HLA	*HLA-A*	100.00	100.00	100.00	100.00	100.00	100.00	100.00	87.50	100.00	100.00	100.00	88.89	100.00	100.00	100.00	100.00	90.91	100.00	100.00	100.00	100.00	88.89	100.00	100.00	98.17
**Original**	***HLA-A***																									
All-HLA	*HLA-A*	100.00	100.00	100.00	100.00	100.00	90.91	100.00	90.00	50.00	100.00	83.33	100.00	100.00	100.00	100.00	88.89	90.91	100.00	100.00	88.89	75.00	100.00	100.00	100.00	94.08
9-HLA	*HLA-A*	100.00	100.00	100.00	100.00	100.00	90.91	100.00	87.50	55.56	100.00	83.33	100.00	100.00	100.00	100.00	88.89	90.91	100.00	100.00	77.78	75.00	100.00	100.00	100.00	93.74
**Original**	***HLA-B***																									
All-HLA	*HLA-B*	88.89	100.00	100.00	100.00	90.00	100.00	100.00	80.00	87.50	100.00	100.00	100.00	100.00	100.00	100.00	100.00	81.82	90.91	100.00	100.00	100.00	100.00	100.00	100.00	96.63
9-HLA	*HLA-B*	88.89	100.00	100.00	100.00	90.00	100.00	100.00	62.50	87.50	100.00	100.00	100.00	100.00	100.00	100.00	100.00	81.82	90.91	100.00	100.00	100.00	100.00	100.00	100.00	95.90
**Original**	***HLA-B***																									
All-HLA	*HLA-B*	100.00	85.71	100.00	100.00	90.00	90.91	100.00	100.00	100.00	100.00	100.00	100.00	100.00	87.50	100.00	100.00	72.73	100.00	100.00	100.00	100.00	100.00	100.00	100.00	96.95
9-HLA	*HLA-B*	100.00	85.71	100.00	100.00	90.00	90.91	100.00	100.00	100.00	100.00	100.00	100.00	100.00	87.50	100.00	100.00	72.73	100.00	100.00	100.00	100.00	100.00	100.00	100.00	96.95
**Original**	***HLA-C***																									
All-HLA	*HLA-C*	77.78	85.71	80.00	90.00	100.00	90.91	100.00	100.00	100.00	100.00	100.00	100.00	90.91	87.50	90.00	88.89	100.00	100.00	100.00	88.89	75.00	88.89	100.00	100.00	93.10
9-HLA	*HLA-C*	77.78	85.71	80.00	90.00	100.00	90.91	100.00	100.00	100.00	100.00	100.00	100.00	90.91	87.50	100.00	88.89	100.00	100.00	100.00	88.89	75.00	88.89	100.00	100.00	93.52
**Original**	***HLA-C***																									
All-HLA	*HLA-C*	88.89	100.00	100.00	100.00	100.00	100.00	100.00	100.00	100.00	83.33	100.00	100.00	100.00	100.00	100.00	88.89	100.00	100.00	100.00	88.89	100.00	100.00	100.00	100.00	97.92
9-HLA	*HLA-C*	88.89	100.00	100.00	100.00	100.00	100.00	100.00	100.00	87.50	83.33	100.00	100.00	100.00	100.00	100.00	88.89	100.00	100.00	100.00	88.89	100.00	100.00	100.00	100.00	97.40
All-HLA	Total %	92.59	95.24	96.67	98.33	96.67	95.45	100.00	90.00	88.43	97.22	97.22	98.15	98.48	95.83	100.00	94.44	89.39	98.48	100.00	92.59	91.67	96.30	100.00	100.00	95.97
9-HLA	Total %	92.59	95.24	96.67	98.33	96.67	95.45	100.00	89.58	88.43	97.22	97.22	98.15	98.48	95.83	100.00	94.44	89.39	98.48	100.00	92.59	91.67	96.30	100.00	100.00	95.95

B HLA Class I

Different HLA Called (out of 11 calls)

Scale: 1 | 2 | 3 | 4 | 5

Different HLA Called

FASTQ	HLA	BRCA_A	CHOR_A	CHOR_B	CHOR_C	ESCA_A	ESCA_B	GBM_A	GBM_B	GBM_C	GBM_D	GBM_E	GBM_F	GBM_G	GBM_H	HGG_A	HGG_B	LGG_A	LGG_B	LIHC_A	LUSC_A	LUSC_B	MISC_A	SARC_A	STAD_A	Total	Ratio
**Original**	***HLA-A***	**1**	**1**	**1**	**1**	**3**	**1**	**1**	**1**	**1**	**1**	**2**	**1**	**1**	**2**	**1**	**1**	**1**	**1**	**1**	**1**	**1**	**2**	**2**	**1**	**30**	**1.25**
All-HLA	*HLA-A*	1	1	1	1	3	1	1	2	1	1	2	2	1	2	1	1	2	1	1	1	1	1	2	1	32	1.33
9-HLA	*HLA-A*	1	1	1	1	3	1	1	2	1	1	2	2	1	2	1	1	2	1	1	1	1	1	2	1	32	1.33
**Original**	***HLA-A***	**1**	**1**	**1**	**3**	**1**	**3**	**1**	**4**	**3**	**1**	**2**	**1**	**2**	**1**	**1**	**2**	**2**	**2**	**1**	**4**	**1**	**1**	**1**	**1**	**41**	**1.71**
All-HLA	*HLA-A*	1	1	2	3	1	2	1	3	3	1	2	1	2	1	1	1	1	2	1	3	2	1	2	1	39	1.63
9-HLA	*HLA-A*	1	1	2	3	1	2	1	3	4	1	2	1	2	1	1	1	1	2	1	3	2	1	2	1	40	1.67
**Original**	***HLA-B***	**1**	**1**	**1**	**2**	**2**	**1**	**1**	**4**	**1**	**1**	**1**	**1**	**2**	**1**	**1**	**1**	**4**	**1**	**1**	**1**	**1**	**1**	**1**	**1**	**33**	**1.38**
All-HLA	*HLA-B*	3	1	1	2	1	1	1	4	2	1	1	1	2	1	1	1	3	2	1	1	1	1	1	1	35	1.46
9-HLA	*HLA-B*	3	1	1	2	1	1	1	3	2	1	1	1	2	1	1	1	3	2	1	1	1	1	1	1	34	1.42
**Original**	***HLA-B***	**1**	**1**	**1**	**1**	**2**	**2**	**1**	**1**	**1**	**1**	**1**	**2**	**3**	**2**	**1**	**1**	**5**	**1**	**1**	**1**	**1**	**1**	**1**	**1**	**34**	**1.42**
All-HLA	*HLA-B*	1	2	1	1	2	3	1	1	1	1	1	1	3	3	1	1	3	1	1	1	1	1	1	2	35	1.46
9-HLA	*HLA-B*	1	2	2	1	2	3	1	1	1	1	1	1	3	3	1	1	3	1	1	1	1	1	1	2	36	1.50
**Original**	***HLA-C***	**4**	**2**	**2**	**2**	**2**	**3**	**1**	**2**	**1**	**1**	**2**	**1**	**1**	**2**	**2**	**1**	**2**	**2**	**1**	**2**	**3**	**3**	**1**	**1**	**44**	**1.83**
All-HLA	*HLA-C*	4	1	3	1	2	4	1	2	1	1	2	1	2	1	3	2	2	2	1	2	2	3	1	1	45	1.88
9-HLA	*HLA-C*	4	1	3	1	2	4	1	2	1	1	2	1	2	1	2	2	2	2	1	2	2	3	1	1	44	1.83
**Original**	***HLA-C***	**4**	**1**	**1**	**1**	**1**	**1**	**1**	**2**	**1**	**1**	**1**	**1**	**3**	**2**	**2**	**2**	**1**	**1**	**1**	**2**	**1**	**1**	**1**	**1**	**34**	**1.42**
All-HLA	*HLA-C*	4	1	1	1	1	1	1	2	1	2	1	1	3	2	1	1	1	1	1	1	1	1	1	1	32	1.33
9-HLA	*HLA-C*	4	1	1	1	1	1	1	2	2	2	1	1	3	2	1	1	1	1	1	1	1	1	1	1	33	1.38
**Original**	**Total**	**12**	**7**	**7**	**10**	**11**	**11**	**6**	**14**	**8**	**6**	**9**	**7**	**12**	**10**	**8**	**8**	**15**	**8**	**6**	**11**	**8**	**9**	**7**	**6**	**216**	**1.50**
All-HLA	Total	14	7	9	9	10	12	6	14	9	7	9	7	13	10	8	7	12	9	6	9	8	8	8	7	218	1.51
9-HLA	Total	14	7	10	9	10	12	6	13	11	7	9	7	13	10	7	7	12	9	6	9	8	8	8	7	219	1.52

Supplementary Figure S3. A] Concordance and B] Number of different HLA called for class I HLA genotypes derived from four tools (ATHLATES, OptiType, Polysolver and seq2HLA) and three data sources.

A HLA Class II

Percent concordance

Scale	20	40	60	80	90	95	100

Concordance

FASTQ	HLA	BRCA_A	CHOR_A	CHOR_B	CHOR_C	ESCA_A	ESCA_B	GBM_A	GBM_B	GBM_C	GBM_D	GBM_E	GBM_F	GBM_G	GBM_H	HGG_A	HGG_B	LGG_A	LGG_B	LIHC_A	LUSC_A	LUSC_B	MISC_A	SARC_A	STAD_A	Total %
**Original**	***HLA-DPA1***																									
All-HLA	*HLA-DPA1*	100	100	100	100	100	100	100	80	100	100	100	100	100	100	100	100	100	100	100	100	100	100	100	100	99.17
9-HLA	*HLA-DPA1*	100	100	100	100	100	100	100	80	100	100	100	100	100	100	100	100	100	100	100	100	100	80	100	100	98.33
**Original**	***HLA-DPA1***																									
All-HLA	*HLA-DPA1*	100	100	100	100	100	100	100	80	100	100	100	100	100	100	100	100	100	100	100	100	100	100	100	100	99.17
9-HLA	*HLA-DPA1*	100	100	100	100	100	100	100	80	100	100	100	100	100	100	100	100	100	100	100	100	100	80	100	100	98.33
**Original**	***HLA-DPB1***																									
All-HLA	*HLA-DPB1*	100	80	60	100	80	80	75	100	100	80	75	80	100	100	80	100	60	100	100	100	60	60	100	80	85.42
9-HLA	*HLA-DPB1*	100	80	60	100	80	80	75	100	100	80	75	80	100	100	80	100	60	100	100	100	60	60	100	80	85.42
**Original**	***HLA-DPB1***																									
All-HLA	*HLA-DPB1*	80	100	40	80	80	60	50	80	80	100	75	80	80	60	60	80	80	100	80	100	80	60	80	80	76.88
9-HLA	*HLA-DPB1*	80	100	40	80	80	60	50	80	60	100	75	80	80	60	60	80	80	100	80	100	80	60	80	80	76.04
**Original**	***HLA-DQA1***																									
All-HLA	*HLA-DQA1*	100	100	80	100	80	100	75	100	100	100	100	100	100	100	100	80	100	100	100	100	100	100	80	66.67	94.24
9-HLA	*HLA-DQA1*	100	100	80	100	80	100	75	100	100	100	100	100	100	100	100	80	100	100	100	100	100	100	80	66.67	94.24
**Original**	***HLA-DQA1***																									
All-HLA	*HLA-DQA1*	80	60	80	100	80	100	75	100	100	100	100	100	100	100	80	60	100	100	100	100	100	100	80	66.67	90.07
9-HLA	*HLA-DQA1*	80	60	80	100	80	100	75	100	100	100	100	100	100	100	80	60	100	100	100	100	100	100	80	66.67	90.07
**Original**	***HLA-DQB1***																									
All-HLA	*HLA-DQB1*	80	100	80	80	80	100	100	80	80	100	75	100	80	60	100	60	75	80	100	80	80	100	100	60	84.58
9-HLA	*HLA-DQB1*	80	100	80	80	80	100	100	80	80	100	75	100	80	60	100	60	75	80	100	80	80	80	100	60	83.75
**Original**	***HLA-DQB1***																									
All-HLA	*HLA-DQB1*	100	60	100	80	60	100	100	80	80	100	75	100	100	60	100	80	75	100	100	80	80	100	100	60	86.25
9-HLA	*HLA-DQB1*	100	60	100	80	60	100	100	80	80	100	75	100	100	60	100	80	75	100	100	80	80	80	100	60	85.42
**Original**	***HLA-DRA***																									
All-HLA	*HLA-DRA*	80	100	100	80	100	100	100	100	100	80	100	100	100	80	100	100	100	100	100	100	80	100	100	100	95.83
9-HLA	*HLA-DRA*	80	100	100	80	100	100	100	100	100	80	100	100	100	80	100	100	100	100	100	100	80	100	100	100	95.83
**Original**	***HLA-DRA***																									
All-HLA	*HLA-DRA*	80	100	100	80	100	100	100	100	100	80	100	100	100	80	100	100	100	100	100	100	80	100	100	100	95.83
9-HLA	*HLA-DRA*	80	100	100	80	100	100	100	100	100	80	100	100	100	80	100	100	100	100	100	100	80	100	100	100	95.83
**Original**	***HLA-DRB1***																									
All-HLA	*HLA-DRB1*	100	100	100	100	100	100	100	100	100	100	100	100	100	100	100	80	80	100	100	100	100	100	100	100	98.33
9-HLA	*HLA-DRB1*	100	100	100	100	100	100	100	100	100	100	100	100	100	100	100	80	80	100	100	100	100	100	100	100	98.33
**Original**	***HLA-DRB1***																									
All-HLA	*HLA-DRB1*	100	80	100	80	80	100	100	100	100	100	50	100	100	100	100	80	100	100	100	100	80	100	100	100	93.75
9-HLA	*HLA-DRB1*	100	80	100	80	80	100	100	100	100	100	50	100	100	100	100	80	100	100	100	100	80	100	100	100	93.75
All-HLA	Total %	91.67	90.00	86.67	90.00	86.67	95.00	89.58	91.67	95.00	95.00	87.50	96.67	96.67	86.67	93.33	85.00	89.17	98.33	98.33	96.67	86.67	93.33	95.00	84.44	91.63
9-HLA	Total %	91.67	90.00	86.67	90.00	86.67	95.00	89.58	91.67	93.33	95.00	87.50	96.67	96.67	86.67	93.33	85.00	89.17	98.33	98.33	96.67	86.67	86.67	95.00	84.44	91.28

B HLA Class II

Different HLA Called (out of 5 calls)

Scale	1	2	3	4

Different HLA Called

FASTQ	HLA	BRCA_A	CHOR_A	CHOR_B	CHOR_C	ESCA_A	ESCA_B	GBM_A	GBM_B	GBM_C	GBM_D	GBM_E	GBM_F	GBM_G	GBM_H	HGG_A	HGG_B	LGG_A	LGG_B	LIHC_A	LUSC_A	LUSC_B	MISC_A	SARC_A	STAD_A	Total	Ratio
**Original**	***HLA-DPA1***	1	1	1	1	1	1	2	1	1	1	1	1	1	1	2	1	2	1	2	1	1	2	2	1	30	1.25
All-HLA	*HLA-DPA1*	1	1	1	1	1	1	2	2	1	1	1	1	1	1	2	1	2	1	2	1	1	2	2	1	31	1.29
9-HLA	*HLA-DPA1*	1	1	1	1	1	1	2	2	1	1	1	1	1	1	2	1	2	1	2	1	1	2	2	1	31	1.29
**Original**	***HLA-DPA1***	1	1	1	1	1	1	1	1	1	1	1	1	1	1	2	1	3	2	2	1	1	2	3	1	32	1.33
All-HLA	*HLA-DPA1*	1	1	1	1	1	1	2	2	1	1	1	1	1	1	2	1	3	2	2	1	1	2	3	1	34	1.42
9-HLA	*HLA-DPA1*	1	1	1	1	1	1	2	2	1	1	1	1	1	1	2	1	3	2	2	1	1	2	3	1	34	1.42
**Original**	***HLA-DPB1***	1	2	2	1	2	1	2	1	1	3	2	2	2	1	2	1	2	2	1	1	2	3	1	2	40	1.67
All-HLA	*HLA-DPB1*	1	2	2	1	4	2	1	2	1	2	1	2	2	1	1	1	3	2	1	1	2	2	1	3	41	1.71
9-HLA	*HLA-DPB1*	1	2	2	1	4	2	1	2	1	2	1	2	2	1	1	1	3	2	1	1	2	1	1	3	40	1.67
**Original**	***HLA-DPB1***	2	1	4	1	4	2	3	3	3	2	2	2	4	3	3	3	3	2	2	1	1	3	2	1	57	2.38
All-HLA	*HLA-DPB1*	1	1	3	2	3	2	2	3	2	2	3	1	3	3	4	2	2	2	1	1	2	2	1	2	50	2.08
9-HLA	*HLA-DPB1*	1	1	3	2	3	2	2	3	1	2	3	1	3	3	4	2	2	2	1	1	2	2	1	2	49	2.04
**Original**	***HLA-DQA1***	1	2	2	3	2	1	2	4	3	3	1	2	2	2	3	4	3	1	2	1	2	1	2	4	53	2.21
All-HLA	*HLA-DQA1*	1	2	2	3	2	1	1	4	3	3	1	2	2	1	3	3	2	1	2	1	2	1	1	3	47	1.96
9-HLA	*HLA-DQA1*	1	2	2	3	2	1	1	4	3	3	1	2	2	1	3	3	2	1	2	1	2	1	1	3	47	1.96
**Original**	***HLA-DQA1***	3	4	3	2	3	2	3	3	2	3	1	1	4	2	4	4	3	2	2	1	2	1	2	4	61	2.54
All-HLA	*HLA-DQA1*	3	2	2	2	3	2	2	3	2	3	1	1	4	1	4	3	2	2	2	1	2	1	1	3	52	2.17
9-HLA	*HLA-DQA1*	3	2	2	2	3	2	2	3	2	3	1	1	4	1	4	3	2	2	2	1	2	1	1	3	52	2.17
**Original**	***HLA-DQB1***	2	1	2	2	2	2	2	4	3	1	2	1	4	4	3	2	3	2	2	4	3	3	3	2	59	2.46
All-HLA	*HLA-DQB1*	3	1	1	2	3	2	2	4	2	1	2	1	3	4	3	2	3	2	2	4	3	3	3	2	58	2.42
9-HLA	*HLA-DQB1*	3	1	1	2	3	2	2	4	2	1	2	1	3	4	3	2	3	2	2	4	3	3	3	2	58	2.42
**Original**	***HLA-DQB1***	1	3	2	2	3	1	2	2	3	2	2	2	2	3	2	4	3	2	2	3	2	3	1	2	54	2.25
All-HLA	*HLA-DQB1*	1	3	2	2	3	1	2	2	2	2	1	2	2	3	2	4	3	2	2	3	2	3	1	2	52	2.17
9-HLA	*HLA-DQB1*	1	3	2	2	3	1	2	2	2	2	1	2	2	3	2	4	3	2	2	3	2	3	1	2	52	2.17
**Original**	***HLA-DRA***	2	1	1	2	1	2	1	1	1	2	1	1	1	2	1	1	1	1	1	1	3	2	1	1	32	1.33
All-HLA	*HLA-DRA*	2	1	1	1	1	2	2	1	1	1	1	1	1	1	1	1	1	1	1	1	1	2	1	1	28	1.17
9-HLA	*HLA-DRA*	2	1	1	1	1	2	2	1	1	1	1	1	1	1	1	1	1	1	1	1	1	2	1	1	28	1.17
**Original**	***HLA-DRA***	2	1	1	2	1	2	2	1	2	2	1	1	1	2	2	1	1	1	1	2	2	2	1	1	35	1.46
All-HLA	*HLA-DRA*	2	1	1	1	1	2	2	1	2	1	1	1	1	2	2	1	1	1	1	2	1	2	1	1	32	1.33
9-HLA	*HLA-DRA*	2	1	1	1	1	2	2	1	2	1	1	1	1	2	2	1	1	1	1	2	1	2	1	1	32	1.33
**Original**	***HLA-DRB1***	2	2	2	1	2	2	1	2	1	2	1	2	1	2	2	2	3	2	2	1	3	2	1	2	43	1.79
All-HLA	*HLA-DRB1*	2	2	2	1	2	2	1	2	1	2	1	2	1	2	2	1	3	2	2	1	3	2	1	2	42	1.75
9-HLA	*HLA-DRB1*	2	2	2	1	2	2	1	2	1	2	1	2	1	2	2	1	3	2	2	1	3	2	1	2	42	1.75
**Original**	***HLA-DRB1***	1	2	1	2	2	1	1	1	1	1	3	1	3	1	2	2	2	1	2	1	2	1	2	2	38	1.58
All-HLA	*HLA-DRB1*	1	1	1	1	3	1	1	1	1	1	3	1	3	1	2	1	2	1	2	1	1	1	2	2	35	1.46
9-HLA	*HLA-DRB1*	1	1	1	1	3	1	1	1	1	1	3	1	3	1	2	1	2	1	2	1	1	1	2	2	35	1.46
**Original**	**Total**	**19**	**21**	**22**	**20**	**24**	**18**	**22**	**24**	**22**	**23**	**18**	**17**	**26**	**24**	**28**	**26**	**29**	**19**	**21**	**18**	**24**	**25**	**21**	**23**	**534**	**1.85**
All-HLA	Total	19	18	19	18	27	19	20	27	19	20	17	16	24	21	28	21	27	19	20	18	21	23	18	23	502	1.74
9-HLA	Total	19	18	19	18	27	19	20	27	18	20	17	16	24	21	28	21	27	19	20	18	21	22	18	23	500	1.74

Supplementary Figure S4. A] Concordance and B] Number of different HLA called for class II HLA genotypes derived from two tools (ATHLATES and seq2HLA) and three data sources.

A ATHLATES - HLA Class I

Percent concordance

Scale: 0 | 20 | 40 | 60 | 80 | 90 | 95 | 100

Concordance

FASTQ	HLA	BRCA_A	CHOR_A	CHOR_B	CHOR_C	ESCA_A	ESCA_B	GBM_A	GBM_B	GBM_C	GBM_D	GBM_E	GBM_F	GBM_G	GBM_H	HGG_A	HGG_B	LGG_A	LGG_B	LIHC_A	LUSC_A	LUSC_B	MISC_A	SARC_A	STAD_A	Total %
**Original**	***HLA-A***																									
All-HLA	*HLA-A*	100.00	100.00	100.00	100.00	100.00	100.00	100.00	100.00	100.00	100.00	100.00	100.00	100.00	100.00	100.00	100.00	100.00	100.00	100.00	100.00	100.00	100.00	100.00	100.00	100.00
9-HLA	*HLA-A*	100.00	100.00	100.00	100.00	100.00	100.00	100.00	100.00	100.00	100.00	100.00	100.00	100.00	100.00	100.00	100.00	100.00	100.00	100.00	100.00	100.00	100.00	100.00	100.00	100.00
**Original**	***HLA-A***																									
All-HLA	*HLA-A*	100.00	100.00	100.00	100.00	100.00	50.00	100.00	100.00	0.00	100.00	50.00	100.00	100.00	100.00	100.00	100.00	100.00	100.00	100.00	100.00	100.00	100.00	100.00	100.00	91.67
9-HLA	*HLA-A*	100.00	100.00	100.00	100.00	100.00	50.00	100.00	100.00	50.00	100.00	50.00	100.00	100.00	100.00	100.00	100.00	100.00	100.00	100.00	50.00	100.00	100.00	100.00	100.00	91.67
**Original**	***HLA-B***																									
All-HLA	*HLA-B*	100.00	100.00	100.00	100.00	100.00	100.00	100.00	100.00	100.00	100.00	100.00	100.00	100.00	100.00	100.00	100.00	100.00	100.00	100.00	100.00	100.00	100.00	100.00	100.00	100.00
9-HLA	*HLA-B*	100.00	100.00	100.00	100.00	100.00	100.00	100.00	50.00	100.00	100.00	100.00	100.00	100.00	100.00	100.00	100.00	100.00	100.00	100.00	100.00	100.00	100.00	100.00	100.00	97.92
**Original**	***HLA-B***																									
All-HLA	*HLA-B*	100.00	100.00	100.00	100.00	100.00	100.00	100.00	100.00	100.00	100.00	100.00	100.00	100.00	100.00	100.00	100.00	100.00	100.00	100.00	100.00	100.00	100.00	100.00	100.00	100.00
9-HLA	*HLA-B*	100.00	100.00	100.00	100.00	100.00	100.00	100.00	100.00	100.00	100.00	100.00	100.00	100.00	100.00	100.00	100.00	100.00	100.00	100.00	100.00	100.00	100.00	100.00	100.00	100.00
**Original**	***HLA-C***																									
All-HLA	*HLA-C*	100.00	100.00	100.00	100.00	100.00	100.00	100.00	100.00	100.00	100.00	100.00	100.00	100.00	100.00	50.00	100.00	100.00	100.00	100.00	100.00	50.00	50.00	100.00	100.00	93.75
9-HLA	*HLA-C*	100.00	100.00	100.00	100.00	100.00	100.00	100.00	100.00	100.00	100.00	100.00	100.00	100.00	100.00	100.00	100.00	100.00	100.00	100.00	100.00	50.00	50.00	100.00	100.00	95.83
**Original**	***HLA-C***																									
All-HLA	*HLA-C*	100.00	100.00	100.00	100.00	100.00	100.00	100.00	100.00	100.00	100.00	100.00	100.00	100.00	100.00	100.00	100.00	100.00	100.00	100.00	100.00	100.00	100.00	100.00	100.00	100.00
9-HLA	*HLA-C*	100.00	100.00	100.00	100.00	100.00	100.00	100.00	100.00	100.00	100.00	100.00	100.00	100.00	100.00	100.00	100.00	100.00	100.00	100.00	100.00	100.00	100.00	100.00	100.00	100.00
All-HLA	Total %	100.00	100.00	100.00	100.00	100.00	91.67	100.00	100.00	83.33	100.00	91.67	100.00	100.00	100.00	91.67	100.00	100.00	100.00	100.00	100.00	91.67	91.67	100.00	100.00	97.57
9-HLA	Total %	100.00	100.00	100.00	100.00	100.00	91.67	100.00	91.67	91.67	100.00	91.67	100.00	100.00	100.00	100.00	100.00	100.00	100.00	100.00	91.67	91.67	91.67	100.00	100.00	97.57

B ATHLATES - HLA Class I

Different HLA Called (out of 2 calls)

Scale: 1 | 2

Different HLA Called

FASTQ	HLA	BRCA_A	CHOR_A	CHOR_B	CHOR_C	ESCA_A	ESCA_B	GBM_A	GBM_B	GBM_C	GBM_D	GBM_E	GBM_F	GBM_G	GBM_H	HGG_A	HGG_B	LGG_A	LGG_B	LIHC_A	LUSC_A	LUSC_B	MISC_A	SARC_A	STAD_A	Total	Ratio
**Original**	***HLA-A***	1	1	1	1	2	1	1	1	1	1	2	1	1	1	1	1	1	1	1	1	1	1	1	1	26	1.08
All-HLA	*HLA-A*	1	1	1	1	2	1	1	1	1	1	2	1	1	1	1	1	1	1	1	1	1	1	1	1	26	1.08
9-HLA	*HLA-A*	1	1	1	1	2	1	1	1	1	1	2	1	1	1	1	1	1	1	1	1	1	1	1	1	26	1.08
**Original**	***HLA-A***	1	1	1	1	1	2	1	1	2	1	2	1	1	1	1	1	1	1	1	2	1	1	1	1	28	1.17
All-HLA	*HLA-A*	1	1	1	1	1	1	1	1	2	1	1	1	1	1	1	1	1	1	1	2	1	1	1	1	26	1.08
9-HLA	*HLA-A*	1	1	1	1	1	1	1	1	2	1	1	1	1	1	1	1	1	1	1	1	1	1	1	1	25	1.04
**Original**	***HLA-B***	1	1	1	1	1	1	1	1	1	1	1	1	1	1	1	1	2	1	1	1	1	1	1	1	25	1.04
All-HLA	*HLA-B*	1	1	1	1	1	1	1	1	1	1	1	1	1	1	1	1	2	1	1	1	1	1	1	1	25	1.04
9-HLA	*HLA-B*	1	1	1	1	1	1	1	2	1	1	1	1	1	1	1	1	2	1	1	1	1	1	1	1	26	1.08
**Original**	***HLA-B***	1	1	1	1	2	1	1	1	1	1	1	1	1	1	1	1	2	1	1	1	1	1	1	1	26	1.08
All-HLA	*HLA-B*	1	1	1	1	2	1	1	1	1	1	1	1	1	1	1	1	2	1	1	1	1	1	1	1	26	1.08
9-HLA	*HLA-B*	1	1	1	1	2	1	1	1	1	1	1	1	1	1	1	1	2	1	1	1	1	1	1	1	26	1.08
**Original**	***HLA-C***	1	1	1	1	2	1	1	1	1	1	1	1	1	1	1	1	1	1	1	1	2	1	1	1	26	1.08
All-HLA	*HLA-C*	1	1	1	1	2	1	1	1	1	1	1	1	1	1	2	1	1	1	1	1	1	2	1	1	27	1.13
9-HLA	*HLA-C*	1	1	1	1	2	1	1	1	1	1	1	1	1	1	1	1	1	1	1	1	1	2	1	1	26	1.08
**Original**	***HLA-C***	1	1	1	1	1	1	1	1	1	1	1	1	1	1	1	1	1	1	1	1	1	1	1	1	24	1.00
All-HLA	*HLA-C*	1	1	1	1	1	1	1	1	1	1	1	1	1	1	1	1	1	1	1	1	1	1	1	1	24	1.00
9-HLA	*HLA-C*	1	1	1	1	1	1	1	1	1	1	1	1	1	1	1	1	1	1	1	1	1	1	1	1	24	1.00
**Original**	**Total**	6	6	6	6	9	7	6	6	7	6	8	6	6	6	6	6	8	6	6	7	7	6	6	6	155	1.08
All-HLA	Total	6	6	6	6	9	6	6	6	7	6	7	6	6	6	7	6	8	6	6	7	6	7	6	6	154	1.07
9-HLA	Total	6	6	6	6	9	6	6	7	7	6	7	6	6	6	6	6	8	6	6	6	6	7	6	6	153	1.06

Supplementary Figure S5. A] Concordance and B] Number of different HLA called for class I HLA genotypes derived using ATHLATES and two data sources (tumor exome and normal exome).

A OptiType - HLA Class I

Percent concordance

Scale	20	40	60	80	90	95	100

Concordance

FASTQ	HLA	BRCA_A	CHOR_A	CHOR_B	CHOR_C	ESCA_A	ESCA_B	GBM_A	GBM_B	GBM_C	GBM_D	GBM_E	GBM_F	GBM_G	GBM_H	HGG_A	HGG_B	LGG_A	LGG_B	LIHC_A	LUSC_A	LUSC_B	MISC_A	SARC_A	STAD_A	Total %
**Original**	***HLA-A***																									
All-HLA	*HLA-A*	100.00	NW	100.00	100.00	100.00	100.00	100.00	100.00	100.00	NW	NW	100.00	100.00	100.00	100.00	100.00	100.00	100.00	100.00	100.00	100.00	100.00	NW	100.00	100.00
9-HLA	*HLA-A*	100.00	NW	100.00	100.00	100.00	100.00	100.00	100.00	100.00	NW	NW	100.00	100.00	100.00	100.00	100.00	100.00	100.00	100.00	100.00	100.00	100.00	NW	100.00	100.00
**Original**	***HLA-A***																									
All-HLA	*HLA-A*	100.00	NW	100.00	100.00	100.00	100.00	100.00	100.00	100.00	NW	NW	100.00	100.00	100.00	100.00	100.00	100.00	100.00	100.00	100.00	100.00	100.00	NW	100.00	100.00
9-HLA	*HLA-A*	100.00	NW	100.00	100.00	100.00	100.00	100.00	100.00	100.00	NW	NW	100.00	100.00	100.00	100.00	100.00	100.00	100.00	100.00	100.00	100.00	100.00	NW	100.00	100.00
**Original**	***HLA-B***																									
All-HLA	*HLA-B*	100.00	NW	100.00	100.00	100.00	100.00	100.00	100.00	100.00	NW	NW	100.00	100.00	100.00	100.00	100.00	100.00	100.00	100.00	100.00	100.00	100.00	NW	100.00	100.00
9-HLA	*HLA-B*	100.00	NW	100.00	100.00	100.00	100.00	100.00	100.00	100.00	NW	NW	100.00	100.00	100.00	100.00	100.00	100.00	100.00	100.00	100.00	100.00	100.00	NW	100.00	100.00
**Original**	***HLA-B***																									
All-HLA	*HLA-B*	100.00	NW	100.00	100.00	100.00	100.00	100.00	100.00	100.00	NW	NW	100.00	100.00	100.00	100.00	100.00	100.00	100.00	100.00	100.00	100.00	100.00	NW	100.00	100.00
9-HLA	*HLA-B*	100.00	NW	100.00	100.00	100.00	100.00	100.00	100.00	100.00	NW	NW	100.00	100.00	100.00	100.00	100.00	100.00	100.00	100.00	100.00	100.00	100.00	NW	100.00	100.00
**Original**	***HLA-C***																									
All-HLA	*HLA-C*	100.00	NW	100.00	100.00	100.00	100.00	100.00	100.00	100.00	NW	NW	100.00	100.00	100.00	100.00	100.00	100.00	100.00	100.00	100.00	100.00	100.00	NW	100.00	100.00
9-HLA	*HLA-C*	100.00	NW	100.00	100.00	100.00	100.00	100.00	100.00	100.00	NW	NW	100.00	100.00	100.00	100.00	100.00	100.00	100.00	100.00	100.00	100.00	100.00	NW	100.00	100.00
**Original**	***HLA-C***																									
All-HLA	*HLA-C*	100.00	NW	100.00	100.00	100.00	100.00	100.00	100.00	100.00	NW	NW	100.00	100.00	100.00	100.00	100.00	100.00	100.00	100.00	100.00	100.00	100.00	NW	100.00	100.00
9-HLA	*HLA-C*	100.00	NW	100.00	100.00	100.00	100.00	100.00	100.00	100.00	NW	NW	100.00	100.00	100.00	100.00	100.00	100.00	100.00	100.00	100.00	100.00	100.00	NW	100.00	100.00
All-HLA	Total %	100.00	NW	100.00	100.00	100.00	100.00	100.00	100.00	100.00	NW	NW	100.00	100.00	100.00	100.00	100.00	100.00	100.00	100.00	100.00	100.00	100.00	NW	100.00	100.00
9-HLA	Total %	100.00	NW	100.00	100.00	100.00	100.00	100.00	100.00	100.00	NW	NW	100.00	100.00	100.00	100.00	100.00	100.00	100.00	100.00	100.00	100.00	100.00	NW	100.00	100.00

B OptiType - HLA Class I

Different HLA Called (out of 3 calls)

Scale	1	2

Different HLA Called

FASTQ	HLA	BRCA_A	CHOR_A	CHOR_B	CHOR_C	ESCA_A	ESCA_B	GBM_A	GBM_B	GBM_C	GBM_D	GBM_E	GBM_F	GBM_G	GBM_H	HGG_A	HGG_B	LGG_A	LGG_B	LIHC_A	LUSC_A	LUSC_B	MISC_A	SARC_A	STAD_A	Total	Ratio
**Original**	***HLA-A***	1	NW	1	1	2	1	1	1	1	NW	NW	1	1	1	1	1	1	1	1	1	1	1	NW	1	21	1.05
All-HLA	*HLA-A*	1	1	1	1	2	1	1	1	1	1	1	1	1	1	1	1	1	1	1	1	1	1	1	1	25	1.04
9-HLA	*HLA-A*	1	1	1	1	2	1	1	1	1	1	1	1	1	1	1	1	1	1	1	1	1	1	1	1	25	1.04
**Original**	***HLA-A***	1	NW	1	1	1	1	1	1	1	NW	NW	1	1	1	1	1	1	1	1	1	1	1	NW	1	20	1.00
All-HLA	*HLA-A*	1	1	1	1	1	1	1	1	1	1	1	1	1	1	1	1	1	1	1	1	1	1	1	1	24	1.00
9-HLA	*HLA-A*	1	1	1	1	1	1	1	1	1	1	1	1	1	1	1	1	1	1	1	1	1	1	1	1	24	1.00
**Original**	***HLA-B***	1	NW	1	1	1	1	1	1	1	NW	NW	1	1	1	1	1	1	1	1	1	1	1	NW	1	20	1.00
All-HLA	*HLA-B*	1	1	1	1	1	1	1	1	1	1	1	1	1	1	1	1	1	1	1	1	1	1	1	1	24	1.00
9-HLA	*HLA-B*	1	1	1	1	1	1	1	1	1	1	1	1	1	1	1	1	1	1	1	1	1	1	1	1	24	1.00
**Original**	***HLA-B***	1	NW	1	1	1	1	1	1	1	NW	NW	1	1	1	1	1	1	1	1	1	1	1	NW	1	20	1.00
All-HLA	*HLA-B*	1	1	1	1	1	1	1	1	1	1	1	1	1	1	1	1	1	1	1	1	1	1	1	1	24	1.00
9-HLA	*HLA-B*	1	1	1	1	1	1	1	1	1	1	1	1	1	1	1	1	1	1	1	1	1	1	1	1	24	1.00
**Original**	***HLA-C***	1	NW	1	1	2	1	1	1	1	NW	NW	1	1	1	1	1	1	1	1	1	1	2	NW	1	22	1.10
All-HLA	*HLA-C*	1	1	1	1	2	1	1	1	1	1	1	1	1	1	1	1	1	1	1	1	1	2	1	1	26	1.08
9-HLA	*HLA-C*	1	1	1	1	2	1	1	1	1	1	1	1	1	1	1	1	1	1	1	1	1	2	1	1	26	1.08
**Original**	***HLA-C***	1	NW	1	1	1	1	1	2	1	NW	NW	1	1	1	1	1	1	1	1	1	1	1	NW	1	21	1.05
All-HLA	*HLA-C*	1	1	1	1	1	1	1	2	1	1	1	1	1	1	1	1	1	1	1	1	1	1	1	1	25	1.04
9-HLA	*HLA-C*	1	1	1	1	1	1	1	2	1	1	1	1	1	1	1	1	1	1	1	1	1	1	1	1	25	1.04
**Original**	**Total**	6	NW	6	6	8	6	6	7	6	NW	NW	6	6	6	6	6	6	6	6	6	6	7	NW	6	124	1.03
All-HLA	Total	6	6	6	6	8	6	6	7	6	6	6	6	6	6	6	6	6	6	6	6	6	7	6	6	148	1.03
9-HLA	Total	6	6	6	6	8	6	6	7	6	6	6	6	6	6	6	6	6	6	6	6	6	7	6	6	148	1.03

Supplementary Figure S6. A] Concordance and B] Number of different HLA called for class I HLA genotypes derived using OptiType and three data sources.

A Polysolver - HLA Class I

Percent concordance

Scale	20	40	60	80	90	95	100

Concordance

FASTQ	HLA	BRCA_A	CHOR_A	CHOR_B	CHOR_C	ESCA_A	ESCA_B	GBM_A	GBM_B	GBM_C	GBM_D	GBM_E	GBM_F	GBM_G	GBM_H	HGG_A	HGG_B	LGG_A	LGG_B	LIHC_A	LUSC_A	LUSC_B	MISC_A	SARC_A	STAD_A	Total %
**Original**	***HLA-A***																									
All-HLA	*HLA-A*	100.00	100.00	100.00	100.00	100.00	100.00	100.00	100.00	100.00	100.00	100.00	100.00	100.00	100.00	100.00	100.00	100.00	100.00	100.00	100.00	100.00	100.00	100.00	100.00	100.00
9-HLA	*HLA-A*	100.00	100.00	100.00	100.00	100.00	100.00	100.00	NW	100.00	100.00	100.00	100.00	100.00	100.00	100.00	100.00	100.00	100.00	100.00	100.00	100.00	100.00	100.00	100.00	100.00
**Original**	***HLA-A***																									
All-HLA	*HLA-A*	100.00	100.00	100.00	100.00	100.00	100.00	100.00	100.00	100.00	100.00	100.00	100.00	100.00	100.00	100.00	100.00	100.00	100.00	100.00	100.00	100.00	100.00	100.00	100.00	100.00
9-HLA	*HLA-A*	100.00	100.00	100.00	100.00	100.00	100.00	100.00	NW	100.00	100.00	100.00	100.00	100.00	100.00	100.00	100.00	100.00	100.00	100.00	100.00	100.00	100.00	100.00	100.00	100.00
**Original**	***HLA-B***																									
All-HLA	*HLA-B*	100.00	100.00	100.00	100.00	100.00	100.00	100.00	100.00	100.00	100.00	100.00	100.00	100.00	100.00	100.00	100.00	66.67	100.00	100.00	100.00	100.00	100.00	100.00	100.00	98.61
9-HLA	*HLA-B*	100.00	100.00	100.00	100.00	100.00	100.00	100.00	NW	100.00	100.00	100.00	100.00	100.00	100.00	100.00	100.00	66.67	100.00	100.00	100.00	100.00	100.00	100.00	100.00	98.55
**Original**	***HLA-B***																									
All-HLA	*HLA-B*	100.00	100.00	100.00	100.00	100.00	100.00	100.00	100.00	100.00	100.00	100.00	100.00	100.00	100.00	100.00	100.00	100.00	100.00	100.00	100.00	100.00	100.00	100.00	100.00	100.00
9-HLA	*HLA-B*	100.00	100.00	100.00	100.00	100.00	100.00	100.00	NW	100.00	100.00	100.00	100.00	100.00	100.00	100.00	100.00	100.00	100.00	100.00	100.00	100.00	100.00	100.00	100.00	100.00
**Original**	***HLA-C***																									
All-HLA	*HLA-C*	100.00	100.00	100.00	100.00	100.00	100.00	100.00	100.00	100.00	100.00	100.00	100.00	100.00	100.00	100.00	100.00	100.00	100.00	100.00	100.00	100.00	100.00	100.00	100.00	100.00
9-HLA	*HLA-C*	100.00	100.00	100.00	100.00	100.00	100.00	100.00	NW	100.00	100.00	100.00	100.00	100.00	100.00	100.00	100.00	100.00	100.00	100.00	100.00	100.00	100.00	100.00	100.00	100.00
**Original**	***HLA-C***																									
All-HLA	*HLA-C*	100.00	100.00	100.00	100.00	100.00	100.00	100.00	100.00	100.00	100.00	100.00	100.00	100.00	100.00	100.00	100.00	100.00	100.00	100.00	100.00	100.00	100.00	100.00	100.00	100.00
9-HLA	*HLA-C*	100.00	100.00	100.00	100.00	100.00	100.00	100.00	NW	100.00	100.00	100.00	100.00	100.00	100.00	100.00	100.00	100.00	100.00	100.00	100.00	100.00	100.00	100.00	100.00	100.00
All-HLA	Total %	100.00	100.00	100.00	100.00	100.00	100.00	100.00	100.00	100.00	100.00	100.00	100.00	100.00	100.00	100.00	100.00	94.44	100.00	100.00	100.00	100.00	100.00	100.00	100.00	99.77
9-HLA	Total %	100.00	100.00	100.00	100.00	100.00	100.00	100.00	NW	100.00	100.00	100.00	100.00	100.00	100.00	100.00	100.00	94.44	100.00	100.00	100.00	100.00	100.00	100.00	100.00	99.76

B Polysolver - HLA Class I

Different HLA Called (out of 3 calls)

Scale	1	2	3

Different HLA Called

FASTQ	HLA	BRCA_A	CHOR_A	CHOR_B	CHOR_C	ESCA_A	ESCA_B	GBM_A	GBM_B	GBM_C	GBM_D	GBM_E	GBM_F	GBM_G	GBM_H	HGG_A	HGG_B	LGG_A	LGG_B	LIHC_A	LUSC_A	LUSC_B	MISC_A	SARC_A	STAD_A	Total	Ratio
**Original**	***HLA-A***	**1**	**1**	**1**	**1**	**2**	**1**	**1**	**1**	**1**	**1**	**1**	**1**	**1**	**1**	**1**	**1**	**1**	**1**	**1**	**1**	**1**	**1**	**1**	**1**	**25**	**1.04**
All-HLA	*HLA-A*	1	1	1	1	2	1	1	1	1	1	1	1	1	1	1	1	1	1	1	1	1	1	1	1	25	1.04
9-HLA	*HLA-A*	1	1	1	1	2	1	1	NW	1	1	1	1	1	1	1	1	1	1	1	1	1	1	1	1	24	1.04
**Original**	***HLA-A***	**1**	**1**	**1**	**2**	**1**	**1**	**1**	**2**	**1**	**1**	**1**	**1**	**2**	**1**	**1**	**1**	**1**	**2**	**1**	**1**	**1**	**1**	**1**	**1**	**28**	**1.17**
All-HLA	*HLA-A*	1	1	1	2	1	1	1	2	1	1	1	1	2	1	1	1	1	2	1	1	1	1	2	1	29	1.21
9-HLA	*HLA-A*	1	1	1	2	1	1	1	NW	1	1	1	1	2	1	1	1	1	2	1	1	1	1	2	1	27	1.17
**Original**	***HLA-B***	**1**	**1**	**1**	**1**	**1**	**1**	**1**	**2**	**1**	**1**	**1**	**1**	**1**	**1**	**1**	**1**	**2**	**1**	**1**	**1**	**1**	**1**	**1**	**1**	**26**	**1.08**
All-HLA	*HLA-B*	2	1	1	1	1	1	1	2	1	1	1	1	1	1	1	1	1	1	1	1	1	1	1	1	26	1.08
9-HLA	*HLA-B*	2	1	1	1	1	1	1	NW	1	1	1	1	1	1	1	1	1	1	1	1	1	1	1	1	24	1.04
**Original**	***HLA-B***	**1**	**1**	**1**	**1**	**2**	**2**	**1**	**1**	**1**	**1**	**1**	**1**	**2**	**1**	**1**	**1**	**1**	**1**	**1**	**1**	**1**	**1**	**1**	**1**	**27**	**1.13**
All-HLA	*HLA-B*	1	1	1	1	2	2	1	1	1	1	1	1	2	1	1	1	1	1	1	1	1	1	2	2	29	1.21
9-HLA	*HLA-B*	1	1	1	1	2	2	1	NW	1	1	1	1	2	1	1	1	1	1	1	1	1	1	2	2	28	1.22
**Original**	***HLA-C***	**1**	**1**	**1**	**1**	**2**	**2**	**1**	**1**	**1**	**1**	**1**	**1**	**1**	**1**	**1**	**1**	**2**	**2**	**1**	**1**	**1**	**1**	**1**	**1**	**28**	**1.17**
All-HLA	*HLA-C*	2	1	1	1	2	2	1	1	1	1	1	1	1	1	1	1	2	2	1	1	1	1	1	1	29	1.21
9-HLA	*HLA-C*	2	1	1	1	2	2	1	NW	1	1	1	1	1	1	1	1	2	2	1	1	1	1	1	1	28	1.22
**Original**	***HLA-C***	**2**	**1**	**1**	**1**	**1**	**1**	**1**	**2**	**1**	**1**	**1**	**1**	**2**	**1**	**1**	**1**	**1**	**1**	**1**	**1**	**1**	**1**	**1**	**1**	**27**	**1.13**
All-HLA	*HLA-C*	1	1	1	1	1	1	1	2	1	1	1	1	2	1	1	1	1	1	1	1	1	1	1	1	26	1.08
9-HLA	*HLA-C*	2	1	1	1	1	1	1	NW	1	1	1	1	2	1	1	1	1	1	1	1	1	1	1	1	25	1.09
**Original**	**Total**	**7**	**6**	**6**	**7**	**9**	**8**	**6**	**9**	**6**	**6**	**6**	**6**	**9**	**6**	**6**	**6**	**8**	**8**	**6**	**6**	**6**	**6**	**6**	**6**	**161**	**1.12**
All-HLA	Total	8	6	6	7	9	8	6	9	6	6	6	6	9	6	6	6	7	8	6	6	6	6	8	7	164	1.14
9-HLA	Total	9	6	6	7	9	8	6	NW	6	6	6	6	9	6	6	6	7	8	6	6	6	6	8	7	156	1.13

Supplementary Figure S7. A] Concordance and B] Number of different HLA called for class I HLA genotypes derived using Polysolver and three data sources.

A seq2HLA - HLA Class I

Percent concordance

Scale	20	40	60	80	90	95	100

Concordance

FASTQ	HLA	BRCA_A	CHOR_A	CHOR_B	CHOR_C	ESCA_A	ESCA_B	GBM_A	GBM_B	GBM_C	GBM_D	GBM_E	GBM_F	GBM_G	GBM_H	HGG_A	HGG_B	LGG_A	LGG_B	LIHC_A	LUSC_A	LUSC_B	MISC_A	SARC_A	STAD_A	Total %
**Original**	***HLA-A***																									
All-HLA	*HLA-A*	100.00	100.00	100.00	100.00	100.00	100.00	100.00	66.67	100.00	100.00	100.00	66.67	100.00	100.00	100.00	100.00	66.67	100.00	100.00	100.00	100.00	66.67	100.00	100.00	94.44
9-HLA	*HLA-A*	100.00	100.00	100.00	100.00	100.00	100.00	100.00	66.67	100.00	100.00	100.00	66.67	100.00	100.00	100.00	100.00	66.67	100.00	100.00	100.00	100.00	66.67	100.00	100.00	94.44
**Original**	***HLA-A***																									
All-HLA	*HLA-A*	100.00	100.00	100.00	100.00	100.00	100.00	100.00	66.67	33.33	100.00	100.00	100.00	100.00	100.00	100.00	66.67	66.67	100.00	100.00	66.67	33.33	100.00	100.00	100.00	88.89
9-HLA	*HLA-A*	100.00	100.00	100.00	100.00	100.00	100.00	100.00	66.67	33.33	100.00	100.00	100.00	100.00	100.00	100.00	66.67	66.67	100.00	100.00	66.67	33.33	100.00	100.00	100.00	88.89
**Original**	***HLA-B***																									
All-HLA	*HLA-B*	66.67	100.00	100.00	100.00	66.67	100.00	100.00	33.33	66.67	100.00	100.00	100.00	100.00	100.00	100.00	100.00	66.67	66.67	100.00	100.00	100.00	100.00	100.00	100.00	90.28
9-HLA	*HLA-B*	66.67	100.00	100.00	100.00	66.67	100.00	100.00	33.33	66.67	100.00	100.00	100.00	100.00	100.00	100.00	100.00	66.67	66.67	100.00	100.00	100.00	100.00	100.00	100.00	90.28
**Original**	***HLA-B***																									
All-HLA	*HLA-B*	100.00	66.67	100.00	100.00	66.67	66.67	100.00	100.00	100.00	100.00	100.00	66.67	100.00	66.67	100.00	100.00	0.00	100.00	100.00	100.00	100.00	100.00	100.00	100.00	88.89
9-HLA	*HLA-B*	100.00	66.67	100.00	100.00	66.67	66.67	100.00	100.00	100.00	100.00	100.00	66.67	100.00	66.67	100.00	100.00	0.00	100.00	100.00	100.00	100.00	100.00	100.00	100.00	88.89
**Original**	***HLA-C***																									
All-HLA	*HLA-C*	33.33	66.67	33.33	66.67	100.00	66.67	100.00	100.00	100.00	100.00	100.00	100.00	66.67	66.67	100.00	66.67	100.00	66.67	100.00	66.67	66.67	100.00	100.00	100.00	81.94
9-HLA	*HLA-C*	33.33	66.67	33.33	66.67	100.00	66.67	100.00	100.00	100.00	100.00	100.00	100.00	66.67	66.67	100.00	66.67	100.00	66.67	100.00	66.67	66.67	100.00	100.00	100.00	81.94
**Original**	***HLA-C***																									
All-HLA	*HLA-C*	66.67	100.00	100.00	100.00	100.00	100.00	100.00	100.00	100.00	66.67	100.00	100.00	100.00	100.00	100.00	66.67	100.00	66.67	100.00	66.67	100.00	100.00	100.00	100.00	93.06
9-HLA	*HLA-C*	66.67	100.00	100.00	100.00	100.00	100.00	100.00	100.00	66.67	66.67	100.00	100.00	100.00	100.00	100.00	66.67	100.00	66.67	100.00	66.67	100.00	100.00	100.00	100.00	91.67
All-HLA	Total %	77.78	88.89	88.89	94.44	88.89	88.89	100.00	77.78	83.33	94.44	100.00	88.89	94.44	88.89	100.00	83.33	66.67	83.33	100.00	83.33	83.33	94.44	100.00	100.00	89.58
9-HLA	Total %	77.78	88.89	88.89	94.44	88.89	88.89	100.00	77.78	77.78	94.44	100.00	88.89	94.44	88.89	100.00	83.33	66.67	83.33	100.00	83.33	83.33	94.44	100.00	100.00	89.35

B seq2HLA - HLA Class I

Different HLA Called (out of 3 calls)

Scale	1	2	3

Different HLA Called

FASTQ	HLA	BRCA_A	CHOR_A	CHOR_B	CHOR_C	ESCA_A	ESCA_B	GBM_A	GBM_B	GBM_C	GBM_D	GBM_E	GBM_F	GBM_G	GBM_H	HGG_A	HGG_B	LGG_A	LGG_B	LIHC_A	LUSC_A	LUSC_B	MISC_A	SARC_A	STAD_A	Total	Ratio
**Original**	***HLA-A***	1	1	1	1	2	1	1	1	1	1	1	1	1	1	1	1	1	1	1	1	1	2	2	1	27	1.13
All-HLA	*HLA-A*	1	1	1	1	2	1	1	2	1	1	1	2	1	1	1	1	2	1	1	1	1	1	2	1	29	1.21
9-HLA	*HLA-A*	1	1	1	1	2	1	1	2	1	1	1	2	1	1	1	1	2	1	1	1	1	1	2	1	29	1.21
**Original**	***HLA-A***	1	1	1	2	1	2	1	3	2	1	1	1	1	1	1	2	2	1	1	3	2	1	1	1	34	1.42
All-HLA	*HLA-A*	1	1	1	2	1	2	1	2	2	1	1	1	1	1	1	1	1	1	1	2	2	1	1	1	30	1.25
9-HLA	*HLA-A*	1	1	1	2	1	2	1	2	2	1	1	1	1	1	1	1	1	1	1	2	2	1	1	1	30	1.25
**Original**	***HLA-B***	1	1	1	2	2	1	1	3	1	1	1	1	2	1	1	1	2	1	1	1	1	1	1	1	30	1.25
All-HLA	*HLA-B*	2	1	1	2	1	1	1	3	2	1	1	1	2	1	1	1	2	2	1	1	1	1	1	1	32	1.33
9-HLA	*HLA-B*	2	1	1	2	1	1	1	3	2	1	1	1	2	1	1	1	2	2	1	1	1	1	1	1	32	1.33
**Original**	***HLA-B***	1	1	1	1	2	1	1	1	1	1	1	1	1	1	1	1	3	1	1	1	1	1	1	1	27	1.13
All-HLA	*HLA-B*	1	2	1	1	1	2	1	1	1	1	1	1	1	2	1	1	2	1	1	1	1	1	1	1	28	1.17
9-HLA	*HLA-B*	1	2	1	1	1	2	1	1	1	1	1	1	1	2	1	1	2	1	1	1	1	1	1	1	28	1.17
**Original**	***HLA-C***	3	2	1	2	2	2	1	2	1	1	1	1	1	2	2	1	2	1	1	2	2	2	1	1	37	1.54
All-HLA	*HLA-C*	2	1	3	1	2	3	1	2	1	1	1	1	2	1	2	2	2	1	1	2	3	2	1	1	39	1.63
9-HLA	*HLA-C*	2	1	3	1	2	3	1	2	1	1	1	1	2	1	2	2	2	1	1	2	3	2	1	1	39	1.63
**Original**	***HLA-C***	2	1	1	1	1	1	1	2	1	1	1	1	2	1	2	2	1	1	1	2	1	1	1	1	30	1.25
All-HLA	*HLA-C*	3	1	1	1	1	1	1	2	1	2	1	1	2	1	2	1	1	1	1	1	1	1	1	1	30	1.25
9-HLA	*HLA-C*	3	1	1	1	1	1	1	2	2	2	1	1	2	1	2	1	1	1	1	1	1	1	1	1	31	1.29
**Original**	**Total**	**9**	**7**	**6**	**9**	**10**	**8**	**6**	**12**	**7**	**6**	**6**	**6**	**8**	**7**	**8**	**8**	**11**	**6**	**6**	**10**	**8**	**8**	**7**	**6**	**185**	**1.28**
All-HLA	Total	10	7	8	8	8	10	6	12	8	7	6	7	9	7	8	7	10	7	6	8	9	7	7	6	188	1.31
9-HLA	Total	10	7	8	8	8	10	6	12	9	7	6	7	9	7	8	7	10	7	6	8	9	7	7	6	189	1.31

Supplementary Figure S8. A] Concordance and B] Number of different HLA called for class I HLA genotypes derived using seq2HLA and three data sources.

A ATHLATES - HLA Class II

Percent concordance
Scale: 20 | 40 | 60 | 80 | 90 | 95 | 100

Concordance

FASTQ	HLA	BRCA_A	CHOR_A	CHOR_B	CHOR_C	ESCA_A	ESCA_B	GBM_A	GBM_B	GBM_C	GBM_D	GBM_E	GBM_F	GBM_G	GBM_H	HGG_A	HGG_B	LGG_A	LGG_B	LIHC_A	LUSC_A	LUSC_B	MISC_A	SARC_A	STAD_A	Total %
**Original**	***HLA1-DPA1***																									
All-HLA	*HLA-DPA1*	100	100	100	100	100	100	100	100	100	100	100	100	100	100	100	100	100	100	100	100	100	100	100	100	100
9-HLA	*HLA-DPA1*	100	100	100	100	100	100	100	100	100	100	100	100	100	100	100	100	100	100	100	100	100	100	100	100	100
**Original**	***HLA-DPA1***																									
All-HLA	*HLA-DPA1*	100	100	100	100	100	100	100	100	100	100	100	100	100	100	100	100	100	100	100	100	100	100	100	100	100
9-HLA	*HLA-DPA1*	100	100	100	100	100	100	100	100	100	100	100	100	100	100	100	100	100	100	100	100	100	100	100	100	100
**Original**	***HLA-DPB1***																									
All-HLA	*HLA-DPB1*	100	100	100	100	100	100	100	100	100	100	100	100	100	100	100	100	100	100	100	100	100	100	100	100	100
9-HLA	*HLA-DPB1*	100	100	100	100	100	100	100	100	100	100	100	100	100	100	100	100	100	100	100	100	100	100	100	100	100
**Original**	***HLA-DPB1***																									
All-HLA	*HLA-DPB1*	100	100	100	100	100	100	100	100	100	100	100	100	100	100	100	100	100	100	100	100	100	100	100	100	100
9-HLA	*HLA-DPB1*	100	100	100	100	100	100	100	100	100	100	100	100	100	100	100	100	100	100	100	100	100	100	100	100	100
**Original**	***HLA-DQA1***																									
All-HLA	*HLA-DQA1*	100	100	100	100	100	100	100	100	100	100	100	100	100	100	100	100	100	100	100	100	100	100	NW	100.00	100
9-HLA	*HLA-DQA1*	100	100	100	100	100	100	100	100	100	100	100	100	100	100	100	100	100	100	100	100	100	100	NW	100.00	100
**Original**	***HLA-DQA1***																									
All-HLA	*HLA-DQA1*	100	100	100	100	100	100	100	100	100	100	100	100	100	100	100	100	100	100	100	100	100	100	NW	100.00	100
9-HLA	*HLA-DQA1*	100	100	100	100	100	100	100	100	100	100	100	100	100	100	100	100	100	100	100	100	100	100	NW	100.00	100
**Original**	***HLA-DQB1***																									
All-HLA	*HLA-DQB1*	100	100	100	100	100	100	100	100	100	100	100	100	100	100	100	100	100	100	100	100	100	100	100	100	100
9-HLA	*HLA-DQB1*	100	100	100	100	100	100	100	100	100	100	100	100	100	100	100	100	100	100	100	100	100	100	100	100	100
**Original**	***HLA-DQB1***																									
All-HLA	*HLA-DQB1*	100	100	100	100	100	100	100	100	100	100	100	100	100	100	100	100	100	100	100	100	100	100	100	100	100
9-HLA	*HLA-DQB1*	100	100	100	100	100	100	100	100	100	100	100	100	100	100	100	100	100	100	100	100	100	100	100	100	100
**Original**	***HLA-DRA***																									
All-HLA	*HLA-DRA*	100	100	100	100	100	100	100	100	100	100	100	100	100	100	100	100	100	100	100	100	100	100	100	100	100
9-HLA	*HLA-DRA*	100	100	100	100	100	100	100	100	100	100	100	100	100	100	100	100	100	100	100	100	100	100	100	100	100
**Original**	***HLA-DRA***																									
All-HLA	*HLA-DRA*	100	100	100	100	100	100	100	100	100	100	100	100	100	100	100	100	100	100	100	100	100	100	100	100	100
9-HLA	*HLA-DRA*	100	100	100	100	100	100	100	100	100	100	100	100	100	100	100	100	100	100	100	100	100	100	100	100	100
**Original**	***HLA-DRB1***																									
All-HLA	*HLA-DRB1*	100	100	100	100	100	100	100	100	100	100	100	100	100	100	100	100	100	100	100	100	100	100	100	100	100
9-HLA	*HLA-DRB1*	100	100	100	100	100	100	100	100	100	100	100	100	100	100	100	100	100	100	100	100	100	100	100	100	100
**Original**	***HLA-DRB1***																									
All-HLA	*HLA-DRB1*	100	100	100	100	100	100	100	100	100	100	100	100	100	100	100	100	100	100	100	100	100	100	100	100	100
9-HLA	*HLA-DRB1*	100	100	100	100	100	100	100	100	100	100	100	100	100	100	100	100	100	100	100	100	100	100	100	100	100
All-HLA	Total %	100	100	100	100	100	100	100	100	100	100	100	100	100	100	100	100	100	100	100	100	100	100	100	100	100
9-HLA	Total %	100	100	100	100	100	100	100	100	100	100	100	100	100	100	100	100	100	100	100	100	100	100	100	100	100

B ATHLATES - HLA Class II

Different HLA Called (out of 2 calls)
Scale: 1 | 2

Different HLA Called

FASTQ	HLA	BRCA_A	CHOR_A	CHOR_B	CHOR_C	ESCA_A	ESCA_B	GBM_A	GBM_B	GBM_C	GBM_D	GBM_E	GBM_F	GBM_G	GBM_H	HGG_A	HGG_B	LGG_A	LGG_B	LIHC_A	LUSC_A	LUSC_B	MISC_A	SARC_A	STAD_A	Total	Ratio
**Original**	***HLA-DPA1***	1	1	1	1	1	1	1	1	1	1	1	1	1	1	1	1	1	1	1	1	1	1	1	1	24	1.00
All-HLA	*HLA-DPA1*	1	1	1	1	1	1	1	1	1	1	1	1	1	1	1	1	1	1	1	1	1	1	1	1	24	1.00
9-HLA	*HLA-DPA1*	1	1	1	1	1	1	1	1	1	1	1	1	1	1	1	1	1	1	1	1	1	1	1	1	24	1.00
**Original**	***HLA-DPA1***	1	1	1	1	1	1	1	1	1	1	1	1	1	1	1	1	2	1	1	1	1	1	1	1	25	1.04
All-HLA	*HLA-DPA1*	1	1	1	1	1	1	1	1	1	1	1	1	1	1	1	1	2	1	1	1	1	1	1	1	25	1.04
9-HLA	*HLA-DPA1*	1	1	1	1	1	1	1	1	1	1	1	1	1	1	1	1	2	1	1	1	1	1	1	1	25	1.04
**Original**	***HLA-DPB1***	1	1	1	1	1	1	1	1	1	1	1	1	1	1	1	1	1	1	1	1	1	1	1	1	24	1.00
All-HLA	*HLA-DPB1*	1	1	1	1	1	1	1	1	1	1	1	1	1	1	1	1	1	1	1	1	1	1	1	1	24	1.00
9-HLA	*HLA-DPB1*	1	1	1	1	1	1	1	1	1	1	1	1	1	1	1	1	1	1	1	1	1	1	1	1	24	1.00
**Original**	***HLA-DPB1***	1	1	1	1	2	1	1	2	1	1	1	1	2	1	1	1	1	1	1	1	1	1	1	1	27	1.13
All-HLA	*HLA-DPB1*	1	1	1	1	2	1	1	2	1	1	1	1	2	1	1	1	1	1	1	1	1	1	1	1	27	1.13
9-HLA	*HLA-DPB1*	1	1	1	1	2	1	1	2	1	1	1	1	2	1	1	1	1	1	1	1	1	1	1	1	27	1.13
**Original**	***HLA-DQA1***	1	1	1	1	1	1	1	2	1	1	1	1	1	1	1	1	1	1	1	1	1	1	NW	1	24	1.04
All-HLA	*HLA-DQA1*	1	1	1	1	1	1	1	2	1	1	1	1	1	1	1	1	1	1	1	1	1	1	NW	1	24	1.04
9-HLA	*HLA-DQA1*	1	1	1	1	1	1	1	2	1	1	1	1	1	1	1	1	1	1	1	1	1	1	NW	1	24	1.04
**Original**	***HLA-DQA1***	1	1	1	1	2	1	1	1	1	1	1	1	1	1	2	1	1	2	1	1	1	1	NW	1	26	1.13
All-HLA	*HLA-DQA1*	1	1	1	1	2	1	1	1	1	1	1	1	1	1	2	1	1	2	1	1	1	1	NW	1	26	1.13
9-HLA	*HLA-DQA1*	1	1	1	1	2	1	1	1	1	1	1	1	1	1	2	1	1	2	1	1	1	1	NW	1	26	1.13
**Original**	***HLA-DQB1***	1	1	1	1	1	2	1	2	1	1	1	1	1	1	1	1	1	1	1	1	1	1	1	1	26	1.08
All-HLA	*HLA-DQB1*	1	1	1	1	1	2	1	2	1	1	1	1	1	1	1	1	1	1	1	1	1	1	1	1	26	1.08
9-HLA	*HLA-DQB1*	1	1	1	1	1	2	1	2	1	1	1	1	1	1	1	1	1	1	1	1	1	1	1	1	26	1.08
**Original**	***HLA-DQB1***	1	1	1	1	2	1	1	2	1	1	1	1	1	1	1	2	1	1	1	1	1	1	1	1	27	1.13
All-HLA	*HLA-DQB1*	1	1	1	1	2	1	1	2	1	1	1	1	1	1	1	2	1	1	1	1	1	1	1	1	27	1.13
9-HLA	*HLA-DQB1*	1	1	1	1	2	1	1	2	1	1	1	1	1	1	1	2	1	1	1	1	1	1	1	1	27	1.13
**Original**	***HLA-DRA***	1	1	1	1	1	1	1	1	1	1	1	1	1	1	1	1	1	1	1	1	1	1	1	1	24	1.00
All-HLA	*HLA-DRA*	1	1	1	1	1	1	1	1	1	1	1	1	1	1	1	1	1	1	1	1	1	1	1	1	24	1.00
9-HLA	*HLA-DRA*	1	1	1	1	1	1	1	1	1	1	1	1	1	1	1	1	1	1	1	1	1	1	1	1	24	1.00
**Original**	***HLA-DRA***	1	1	1	1	1	1	1	1	1	1	1	1	1	1	1	1	1	1	1	1	1	1	1	1	24	1.00
All-HLA	*HLA-DRA*	1	1	1	1	1	1	1	1	1	1	1	1	1	1	1	1	1	1	1	1	1	1	1	1	24	1.00
9-HLA	*HLA-DRA*	1	1	1	1	1	1	1	1	1	1	1	1	1	1	1	1	1	1	1	1	1	1	1	1	24	1.00
**Original**	***HLA-DRB1***	1	2	1	1	1	1	1	1	1	1	1	1	1	1	2	1	2	1	1	1	1	1	1	1	27	1.13
All-HLA	*HLA-DRB1*	1	2	1	1	1	1	1	1	1	1	1	1	1	1	2	1	2	1	1	1	1	1	1	1	27	1.13
9-HLA	*HLA-DRB1*	1	2	1	1	1	1	1	1	1	1	1	1	1	1	2	1	2	1	1	1	1	1	1	1	27	1.13
**Original**	***HLA-DRB1***	1	1	1	1	2	1	1	1	1	1	1	1	1	1	1	1	2	1	1	1	1	1	1	1	26	1.08
All-HLA	*HLA-DRB1*	1	1	1	1	2	1	1	1	1	1	1	1	1	1	1	1	2	1	1	1	1	1	1	1	26	1.08
9-HLA	*HLA-DRB1*	1	1	1	1	2	1	1	1	1	1	1	1	1	1	1	1	2	1	1	1	1	1	1	1	26	1.08
**Original**	**Total**	12	13	12	12	16	13	12	16	12	12	12	12	13	12	14	13	15	13	12	12	12	12	10	12	304	1.06
All-HLA	Total	12	13	12	12	16	13	12	16	12	12	12	12	13	12	14	13	15	13	12	12	12	12	10	12	304	1.06
9-HLA	Total	12	13	12	12	16	13	12	16	12	12	12	12	13	12	14	13	15	13	12	12	12	12	10	12	304	1.06

Supplementary Figure S9. A] Concordance and B] Number of different HLA called for class II HLA genotypes derived using ATHLATES and two data sources (tumor exome and normal exome).

A seq2HLA - HLA Class II

Percent concordance

Scale: 0 | 20 | 40 | 60 | 80 | 90 | 95 | 100

Concordance

FASTQ	HLA	BRCA_A	CHOR_A	CHOR_B	CHOR_C	ESCA_A	ESCA_B	GBM_A	GBM_B	GBM_C	GBM_D	GBM_E	GBM_F	GBM_G	GBM_H	HGG_A	HGG_B	LGG_A	LGG_B	LIHC_A	LUSC_A	LUSC_B	MISC_A	SARC_A	STAD_A	Total %
**Original**	***HLA-DPA1***																									
All-HLA	*HLA-DPA1*	100.00	100.00	100.00	100.00	100.00	100.00	100.00	66.67	100.00	100.00	100.00	100.00	100.00	100.00	100.00	100.00	100.00	100.00	100.00	100.00	100.00	66.67	100.00	100.00	97.22
9-HLA	*HLA-DPA1*	100.00	100.00	100.00	100.00	100.00	100.00	100.00	66.67	100.00	100.00	100.00	100.00	100.00	100.00	100.00	100.00	100.00	100.00	100.00	100.00	100.00	66.67	100.00	100.00	97.22
**Original**	***HLA-DPA1***																									
All-HLA	*HLA-DPA1*	100.00	100.00	100.00	100.00	100.00	100.00	100.00	66.67	100.00	100.00	100.00	100.00	100.00	100.00	100.00	100.00	100.00	100.00	100.00	100.00	100.00	66.67	100.00	100.00	97.22
9-HLA	*HLA-DPA1*	100.00	100.00	100.00	100.00	100.00	100.00	100.00	66.67	100.00	100.00	100.00	100.00	100.00	100.00	100.00	100.00	100.00	100.00	100.00	100.00	100.00	66.67	100.00	100.00	97.22
**Original**	***HLA-DPB1***																									
All-HLA	*HLA-DPB1*	100.00	66.67	33.33	100.00	33.33	66.67	50.00	100.00	100.00	66.67	50.00	66.67	100.00	100.00	66.67	100.00	33.33	100.00	100.00	100.00	33.33	33.33	100.00	66.67	73.61
9-HLA	*HLA-DPB1*	100.00	66.67	33.33	100.00	33.33	66.67	50.00	100.00	100.00	66.67	50.00	66.67	100.00	100.00	66.67	100.00	33.33	100.00	100.00	100.00	33.33	33.33	100.00	66.67	73.61
**Original**	***HLA-DPB1***																									
All-HLA	*HLA-DPB1*	66.67	100.00	0.00	66.67	66.67	33.33	0.00	66.67	66.67	100.00	50.00	66.67	66.67	33.33	33.33	66.67	66.67	100.00	66.67	100.00	66.67	33.33	66.67	66.67	60.42
9-HLA	*HLA-DPB1*	66.67	100.00	0.00	66.67	66.67	33.33	0.00	66.67	33.33	100.00	50.00	66.67	66.67	33.33	33.33	66.67	66.67	100.00	66.67	100.00	66.67	33.33	66.67	66.67	59.03
**Original**	***HLA-DQA1***																									
All-HLA	*HLA-DQA1*	100.00	100.00	66.67	100.00	66.67	100.00	50.00	100.00	100.00	100.00	100.00	100.00	100.00	NW	100.00	66.67	100.00	100.00	100.00	100.00	100.00	100.00	100.00	50.00	91.30
9-HLA	*HLA-DQA1*	100.00	100.00	66.67	100.00	66.67	100.00	50.00	100.00	100.00	100.00	100.00	100.00	100.00	NW	100.00	66.67	100.00	100.00	100.00	100.00	100.00	100.00	100.00	50.00	91.30
**Original**	***HLA-DQA1***																									
All-HLA	*HLA-DQA1*	66.67	33.33	66.67	100.00	66.67	100.00	50.00	100.00	100.00	100.00	100.00	100.00	100.00	NW	66.67	33.33	100.00	100.00	100.00	100.00	100.00	100.00	100.00	50.00	84.06
9-HLA	*HLA-DQA1*	66.67	33.33	66.67	100.00	66.67	100.00	50.00	100.00	100.00	100.00	100.00	100.00	100.00	NW	66.67	33.33	100.00	100.00	100.00	100.00	100.00	100.00	100.00	50.00	84.06
**Original**	***HLA-DQB1***																									
All-HLA	*HLA-DQB1*	66.67	100.00	66.67	66.67	66.67	100.00	100.00	66.67	66.67	100.00	50.00	100.00	66.67	33.33	100.00	33.33	66.67	66.67	100.00	66.67	66.67	100.00	100.00	33.33	74.31
9-HLA	*HLA-DQB1*	66.67	100.00	66.67	66.67	66.67	100.00	100.00	66.67	66.67	100.00	50.00	100.00	66.67	33.33	100.00	33.33	66.67	66.67	100.00	66.67	66.67	66.67	100.00	33.33	72.92
**Original**	***HLA-DQB1***																									
All-HLA	*HLA-DQB1*	100.00	33.33	100.00	66.67	33.33	100.00	100.00	100.00	100.00	100.00	50.00	100.00	100.00	33.33	100.00	33.33	66.67	100.00	100.00	66.67	66.67	100.00	100.00	33.33	78.47
9-HLA	*HLA-DQB1*	100.00	33.33	100.00	66.67	33.33	100.00	100.00	100.00	100.00	100.00	50.00	100.00	100.00	33.33	100.00	33.33	66.67	100.00	100.00	66.67	66.67	66.67	100.00	33.33	77.08
**Original**	***HLA-DRA***																									
All-HLA	*HLA-DRA*	66.67	100.00	100.00	66.67	100.00	100.00	100.00	100.00	100.00	66.67	100.00	100.00	100.00	66.67	100.00	100.00	100.00	100.00	100.00	100.00	66.67	100.00	100.00	100.00	93.06
9-HLA	*HLA-DRA*	66.67	100.00	100.00	66.67	100.00	100.00	100.00	100.00	100.00	66.67	100.00	100.00	100.00	66.67	100.00	100.00	100.00	100.00	100.00	100.00	66.67	100.00	100.00	100.00	93.06
**Original**	***HLA-DRA***																									
All-HLA	*HLA-DRA*	66.67	100.00	100.00	66.67	100.00	100.00	100.00	100.00	100.00	66.67	100.00	100.00	100.00	66.67	100.00	100.00	100.00	100.00	100.00	100.00	66.67	100.00	100.00	100.00	93.06
9-HLA	*HLA-DRA*	66.67	100.00	100.00	66.67	100.00	100.00	100.00	100.00	100.00	66.67	100.00	100.00	100.00	66.67	100.00	100.00	100.00	100.00	100.00	100.00	66.67	100.00	100.00	100.00	93.06
**Original**	***HLA-DRB1***																									
All-HLA	*HLA-DRB1*	100.00	100.00	100.00	100.00	100.00	100.00	100.00	100.00	100.00	100.00	100.00	100.00	100.00	100.00	100.00	66.67	66.67	100.00	100.00	100.00	100.00	100.00	100.00	100.00	97.22
9-HLA	*HLA-DRB1*	100.00	100.00	100.00	100.00	100.00	100.00	100.00	100.00	100.00	100.00	100.00	100.00	100.00	100.00	100.00	66.67	66.67	100.00	100.00	100.00	100.00	100.00	100.00	100.00	97.22
**Original**	***HLA-DRB1***																									
All-HLA	*HLA-DRB1*	100.00	66.67	100.00	100.00	66.67	100.00	100.00	100.00	100.00	100.00	0.00	100.00	100.00	100.00	100.00	66.67	100.00	100.00	100.00	100.00	66.67	100.00	100.00	100.00	90.28
9-HLA	*HLA-DRB1*	100.00	66.67	100.00	100.00	66.67	100.00	100.00	100.00	100.00	100.00	0.00	100.00	100.00	100.00	100.00	66.67	100.00	100.00	100.00	100.00	66.67	100.00	100.00	100.00	90.28
All-HLA	Total %	86.11	83.33	77.78	86.11	75.00	91.67	79.17	88.89	94.44	91.67	75.00	94.44	94.44	73.33	88.89	72.22	83.33	97.22	97.22	94.44	77.78	83.33	97.22	75.00	85.85
9-HLA	Total %	86.11	83.33	77.78	86.11	75.00	91.67	79.17	88.89	91.67	91.67	75.00	94.44	94.44	73.33	88.89	72.22	83.33	97.22	97.22	94.44	77.78	77.78	97.22	75.00	85.50

B seq2HLA - HLA Class II

Different HLA Called (out of 3 calls)

Scale: 1 | 2 | 3

Different HLA Called

FASTQ	HLA	BRCA_A	CHOR_A	CHOR_B	CHOR_C	ESCA_A	ESCA_B	GBM_A	GBM_B	GBM_C	GBM_D	GBM_E	GBM_F	GBM_G	GBM_H	HGG_A	HGG_B	LGG_A	LGG_B	LIHC_A	LUSC_A	LUSC_B	MISC_A	SARC_A	STAD_A	Total	Ratio
**Original**	***HLA-DPA1***	**1**	**1**	**1**	**1**	**1**	1	**1**	**1**	**1**	**1**	**1**	**1**	**1**	**1**	**2**	**1**	**2**	**1**	**2**	**1**	**1**	**2**	**2**	**1**	**29**	**1.21**
All-HLA	*HLA-DPA1*	1	1	1	1	1	1	2	2	1	1	1	1	1	1	2	1	2	1	2	1	1	2	2	1	31	1.29
9-HLA	*HLA-DPA1*	1	1	1	1	1	1	2	2	1	1	1	1	1	1	2	1	2	1	2	1	1	2	2	1	31	1.29
**Original**	***HLA-DPA1***	**1**	**1**	**1**	**1**	**1**	1	**1**	**1**	**1**	**1**	**1**	**1**	**1**	**1**	**2**	**1**	**2**	**1**	**2**	**1**	**1**	**2**	**2**	**1**	**29**	**1.21**
All-HLA	*HLA-DPA1*	1	1	1	1	1	1	2	2	1	1	1	1	1	1	2	1	2	1	2	1	1	2	2	1	31	1.29
9-HLA	*HLA-DPA1*	1	1	1	1	1	1	2	2	1	1	1	1	1	1	2	1	2	1	2	1	1	2	2	1	31	1.29
**Original**	***HLA-DPB1***	**1**	**2**	**2**	**1**	**1**	1	**2**	**1**	**1**	**3**	**2**	**2**	**2**	**1**	**2**	**1**	**2**	**2**	**1**	**1**	**2**	**3**	**1**	**2**	**39**	**1.63**
All-HLA	*HLA-DPB1*	1	2	2	1	3	2	1	1	1	2	1	2	2	1	1	1	3	2	1	1	1	2	1	3	38	1.58
9-HLA	*HLA-DPB1*	1	2	2	1	3	2	1	1	1	2	1	2	2	1	1	1	3	2	1	1	1	1	1	3	37	1.54
**Original**	***HLA-DPB1***	**2**	**1**	**3**	**1**	**3**	2	**1**	**2**	**3**	**2**	**2**	**2**	**3**	**2**	**3**	**2**	**2**	**2**	**2**	**1**	**1**	**3**	**2**	**1**	**48**	**2.00**
All-HLA	*HLA-DPB1*	1	1	3	2	2	2	2	2	2	2	3	1	2	2	3	1	1	2	1	1	2	2	1	2	43	1.79
9-HLA	*HLA-DPB1*	1	1	3	2	2	2	2	2	1	2	3	1	2	2	3	1	1	2	1	1	2	2	1	2	42	1.75
**Original**	***HLA-DQA1***	**1**	**2**	**2**	**2**	**2**	1	**2**	**2**	**2**	**2**	**1**	**1**	**2**	**2**	**2**	**3**	**2**	**1**	**1**	**1**	**1**	**1**	**2**	**3**	**41**	**1.71**
All-HLA	*HLA-DQA1*	1	2	1	2	2	1	1	2	2	2	1	1	2	NW	2	2	1	1	1	1	1	1	1	2	33	1.43
9-HLA	*HLA-DQA1*	1	2	1	2	2	1	1	2	2	2	1	1	2	NW	2	2	1	1	1	1	1	1	1	2	33	1.43
**Original**	***HLA-DQA1***	**2**	**3**	**2**	**2**	**2**	2	**2**	**3**	**2**	**2**	**1**	**1**	**3**	**2**	**2**	**3**	**2**	**1**	**1**	**1**	**1**	**1**	**2**	**3**	**46**	**1.92**
All-HLA	*HLA-DQA1*	3	1	1	2	2	2	1	3	2	2	1	1	3	NW	2	2	1	1	1	1	1	1	1	2	37	1.61
9-HLA	*HLA-DQA1*	3	1	1	2	2	2	1	3	2	2	1	1	3	NW	2	2	1	1	1	1	1	1	1	2	37	1.61
**Original**	***HLA-DQB1***	**2**	**1**	**2**	**2**	**2**	1	**1**	**3**	**2**	**1**	**1**	**1**	**3**	**3**	**2**	**2**	**3**	**2**	**2**	**3**	**2**	**2**	**2**	**2**	**47**	**1.96**
All-HLA	*HLA-DQB1*	2	1	1	2	3	1	1	2	2	1	2	1	2	3	2	2	3	2	2	3	2	2	2	2	46	1.92
9-HLA	*HLA-DQB1*	2	1	1	2	3	1	1	2	2	1	2	1	2	3	2	2	3	2	2	3	2	2	2	2	46	1.92
**Original**	***HLA-DQB1***	**1**	**2**	**2**	**2**	**2**	1	**1**	**2**	**3**	**2**	**2**	**1**	**2**	**3**	**2**	**2**	**3**	**1**	**2**	**3**	**2**	**2**	**1**	**2**	**46**	**1.92**
All-HLA	*HLA-DQB1*	1	2	2	2	2	1	1	1	2	2	1	1	2	3	2	2	3	1	2	3	2	2	1	2	43	1.79
9-HLA	*HLA-DQB1*	1	2	2	2	2	1	1	1	2	2	1	1	2	3	2	2	3	1	2	3	2	2	1	2	43	1.79
**Original**	***HLA-DRA***	**2**	**1**	**1**	**2**	**1**	2	**1**	**1**	**1**	**2**	**1**	**1**	**1**	**2**	**1**	**1**	**1**	**1**	**1**	**1**	**2**	**1**	**1**	**3**	**32**	**1.33**
All-HLA	*HLA-DRA*	2	1	1	1	1	2	2	1	1	1	1	1	1	1	1	1	1	1	1	1	1	1	1	3	29	1.21
9-HLA	*HLA-DRA*	2	1	1	1	1	2	2	1	1	1	1	1	1	1	1	1	1	1	1	1	1	1	1	3	29	1.21
**Original**	***HLA-DRA***	**2**	**1**	**1**	**2**	**1**	2	**1**	**1**	**1**	**2**	**1**	**1**	**1**	**2**	**1**	**1**	**1**	**1**	**1**	**1**	**2**	**1**	**1**	**3**	**32**	**1.33**
All-HLA	*HLA-DRA*	2	1	1	1	1	2	2	1	1	1	1	1	1	1	1	1	1	1	1	1	1	1	1	3	29	1.21
9-HLA	*HLA-DRA*	2	1	1	1	1	2	2	1	1	1	1	1	1	1	1	1	1	1	1	1	1	1	1	3	29	1.21
**Original**	***HLA-DRB1***	**2**	**1**	**2**	**1**	**2**	2	**1**	**2**	**1**	**2**	**1**	**2**	**1**	**2**	**1**	**2**	**2**	**2**	**2**	**1**	**3**	**2**	**1**	**2**	**40**	**1.67**
All-HLA	*HLA-DRB1*	2	1	2	1	2	2	1	2	1	2	1	2	1	2	1	1	2	2	2	1	3	2	1	2	39	1.63
9-HLA	*HLA-DRB1*	2	1	2	1	2	2	1	2	1	2	1	2	1	2	1	1	2	2	2	1	3	2	1	2	39	1.63
**Original**	***HLA-DRB1***	**1**	**2**	**1**	**2**	**1**	1	**1**	**1**	**1**	**1**	**2**	**1**	**2**	**1**	**1**	**2**	**1**	**1**	**2**	**1**	**2**	**1**	**2**	**1**	**32**	**1.33**
All-HLA	*HLA-DRB1*	1	1	1	1	2	1	1	1	1	1	2	1	2	1	1	1	1	1	2	1	1	1	2	1	29	1.21
9-HLA	*HLA-DRB1*	1	1	1	1	2	1	1	1	1	1	2	1	2	1	1	1	1	1	2	1	1	1	2	1	29	1.21
**Original**	**Total**	**18**	**18**	**20**	**19**	**19**	**17**	**15**	**20**	**19**	**21**	**16**	**15**	**22**	**22**	**21**	**21**	**23**	**16**	**19**	**16**	**20**	**21**	**19**	**24**	**461**	**1.60**
All-HLA	Total	18	15	17	17	22	18	17	20	17	18	16	14	20	16	20	16	21	16	18	16	17	19	16	24	428	1.49
9-HLA	Total	18	15	17	17	22	18	17	20	16	18	16	14	20	16	20	16	21	16	18	16	17	18	16	24	426	1.48

Supplementary Figure S10. A] Concordance and B] Number of different HLA called for class II HLA genotypes derived using seq2HLA and three data sources.
