# Supplementary figures and images for: Evaluation of HLA Region-Specific High-Throughput Sequencing FASTQ Reads Combined with Ensemble HLA-Typing Tools for Rapid and High-Confidence HLA Typing
Source: Biology (Basel). 2025 Dec 1;14(12):1717. doi: 10.3390/biology14121717 (PMC12731231; doi:10.3390/biology14121717)

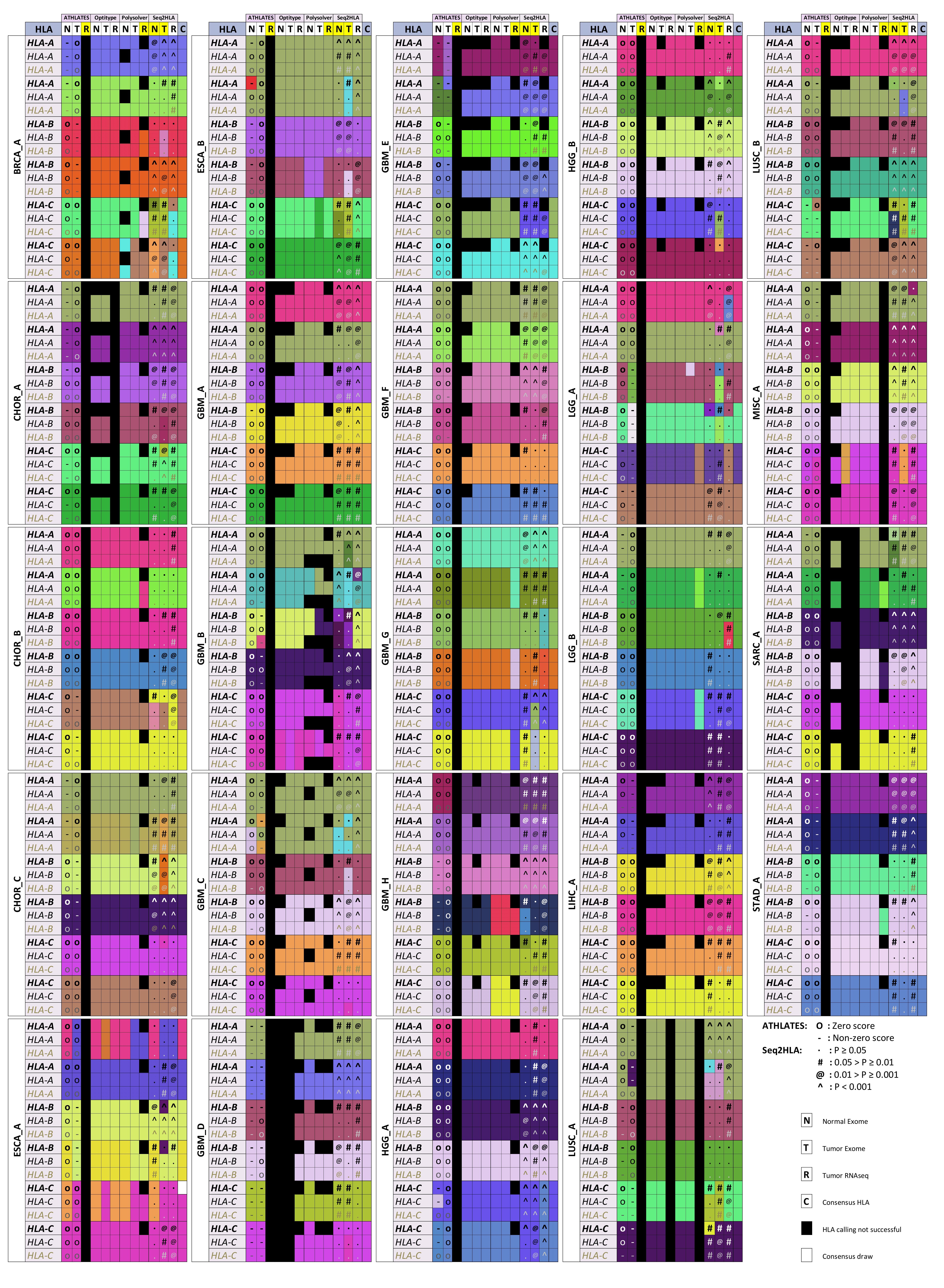

Supplement: Supplementary file 1 [file biology-14-01717-s001.zip › SupplementaryFigure-S1.jpg]

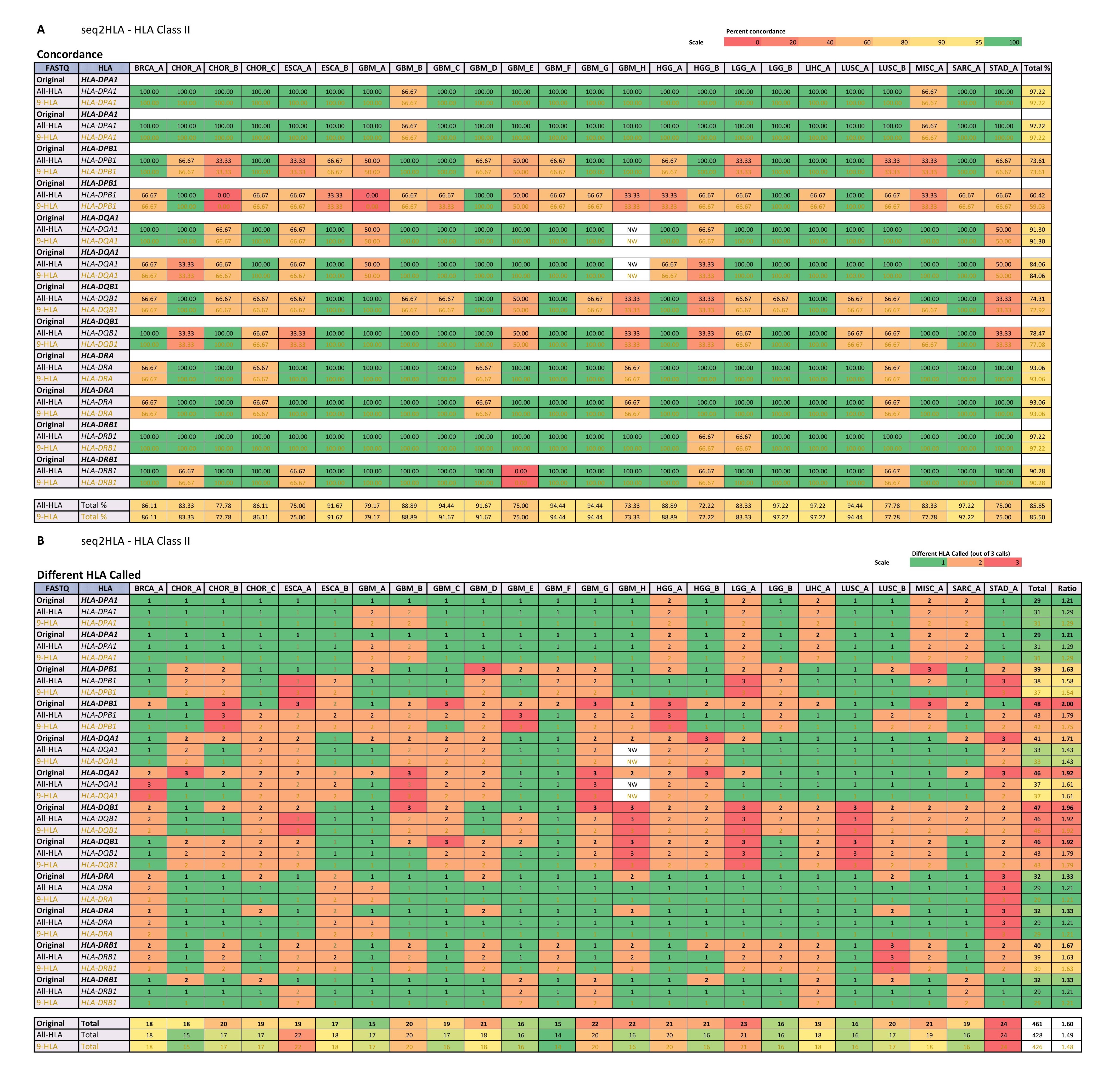

Supplement: Supplementary file 1 [file biology-14-01717-s001.zip › SupplementaryFigure-S10.jpg]

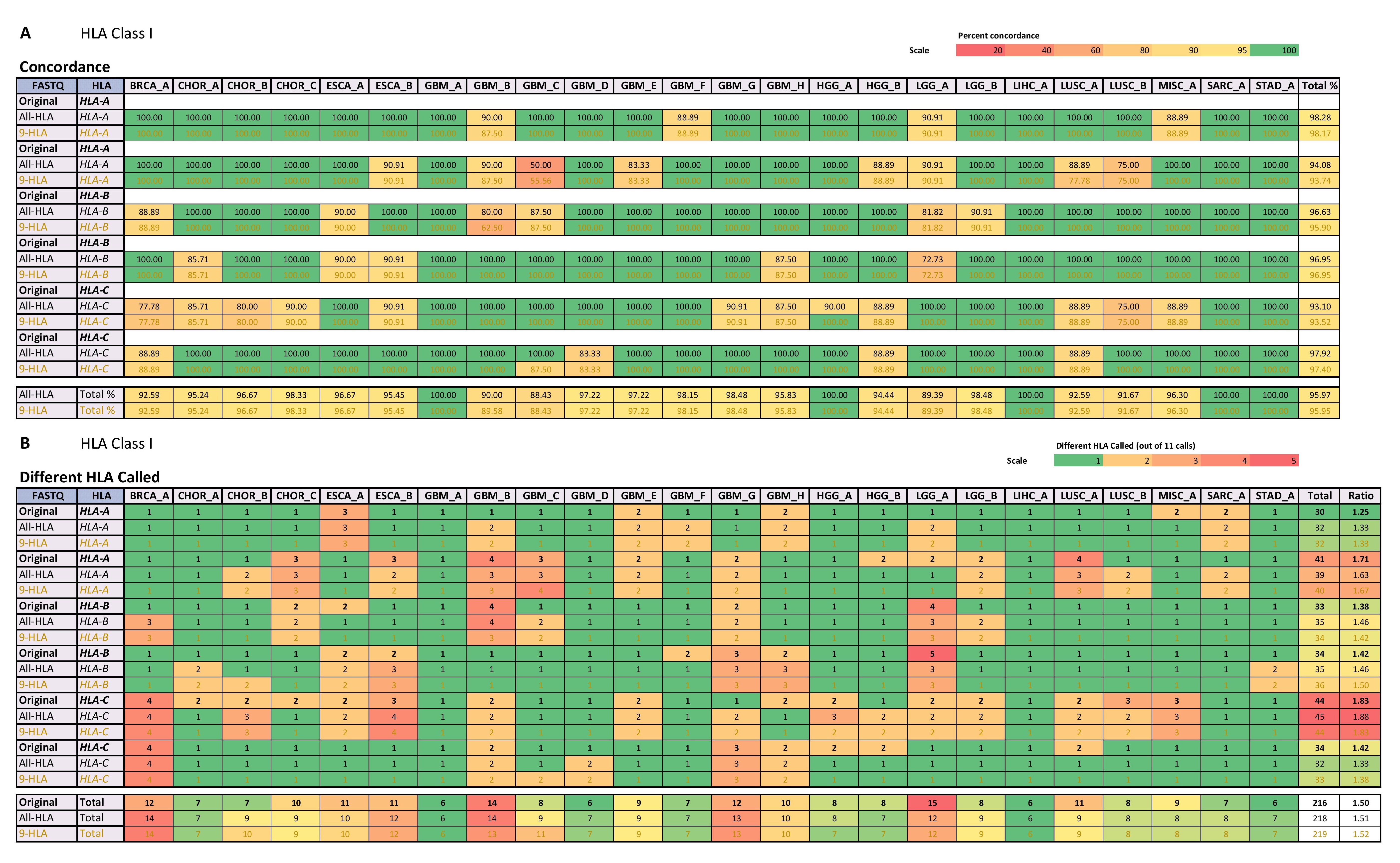

Supplement: Supplementary file 1 [file biology-14-01717-s001.zip › SupplementaryFigure-S3.jpg]

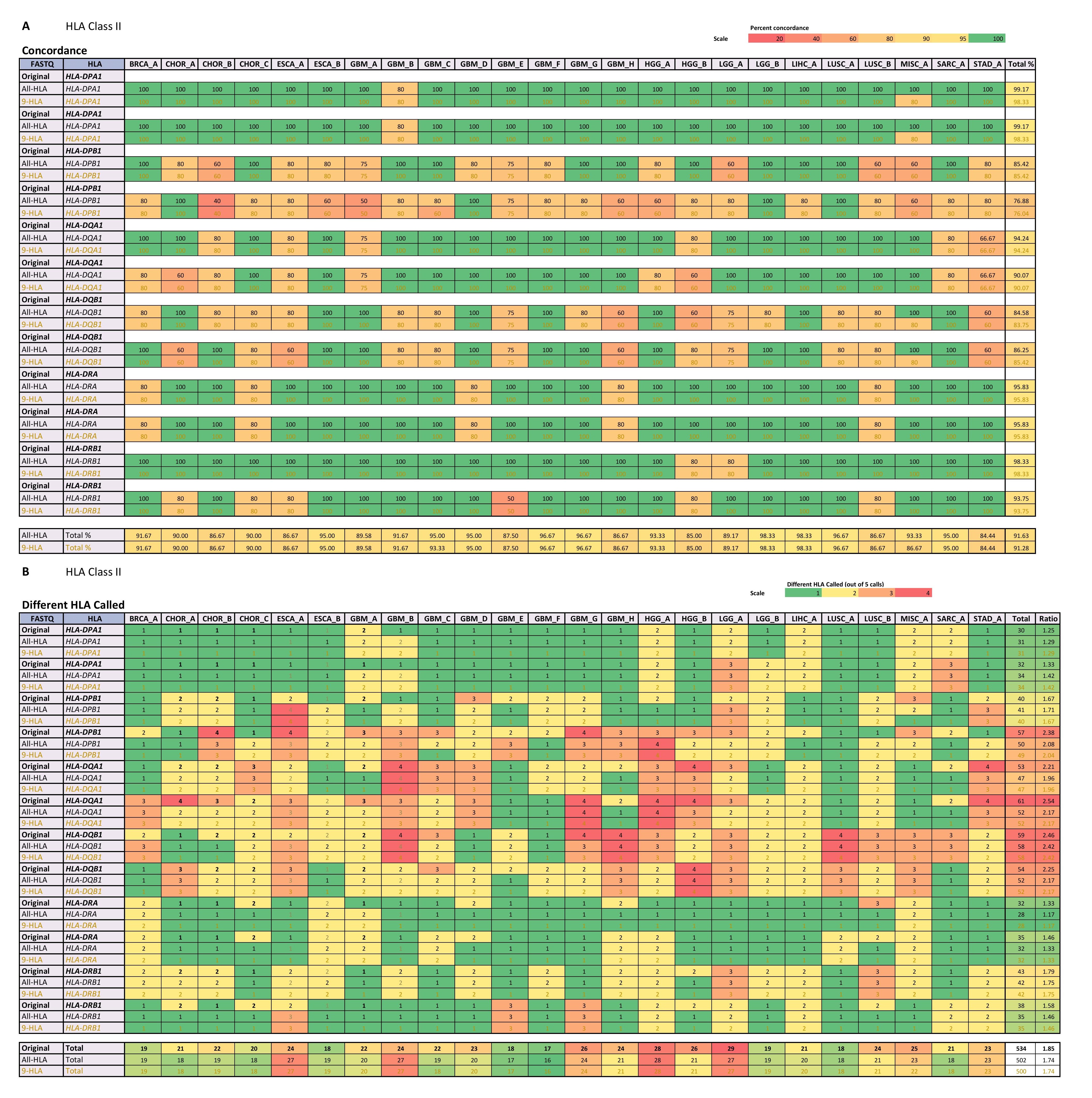

Supplement: Supplementary file 1 [file biology-14-01717-s001.zip › SupplementaryFigure-S4.jpg]

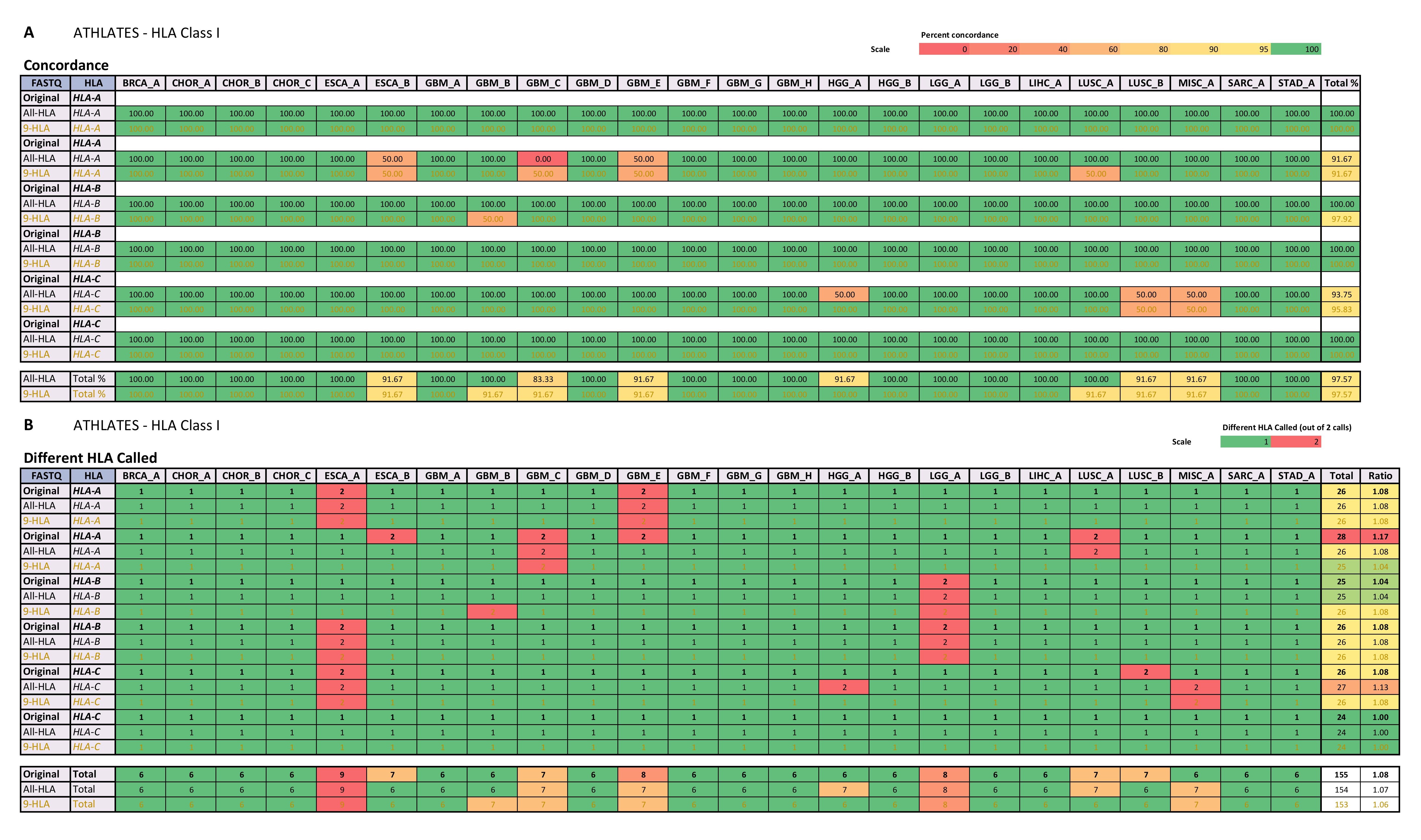

Supplement: Supplementary file 1 [file biology-14-01717-s001.zip › SupplementaryFigure-S5.jpg]

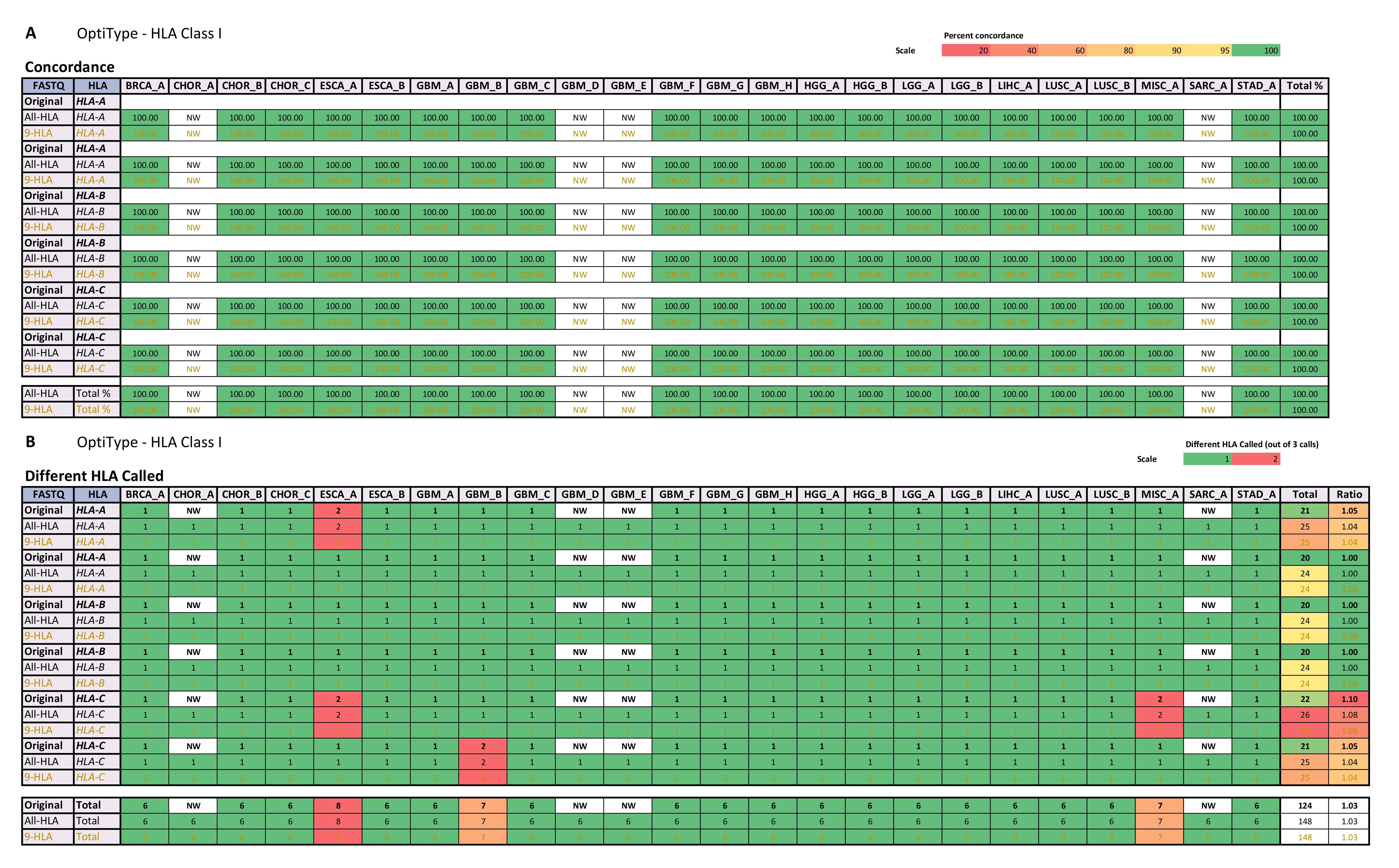

Supplement: Supplementary file 1 [file biology-14-01717-s001.zip › SupplementaryFigure-S6.jpg]

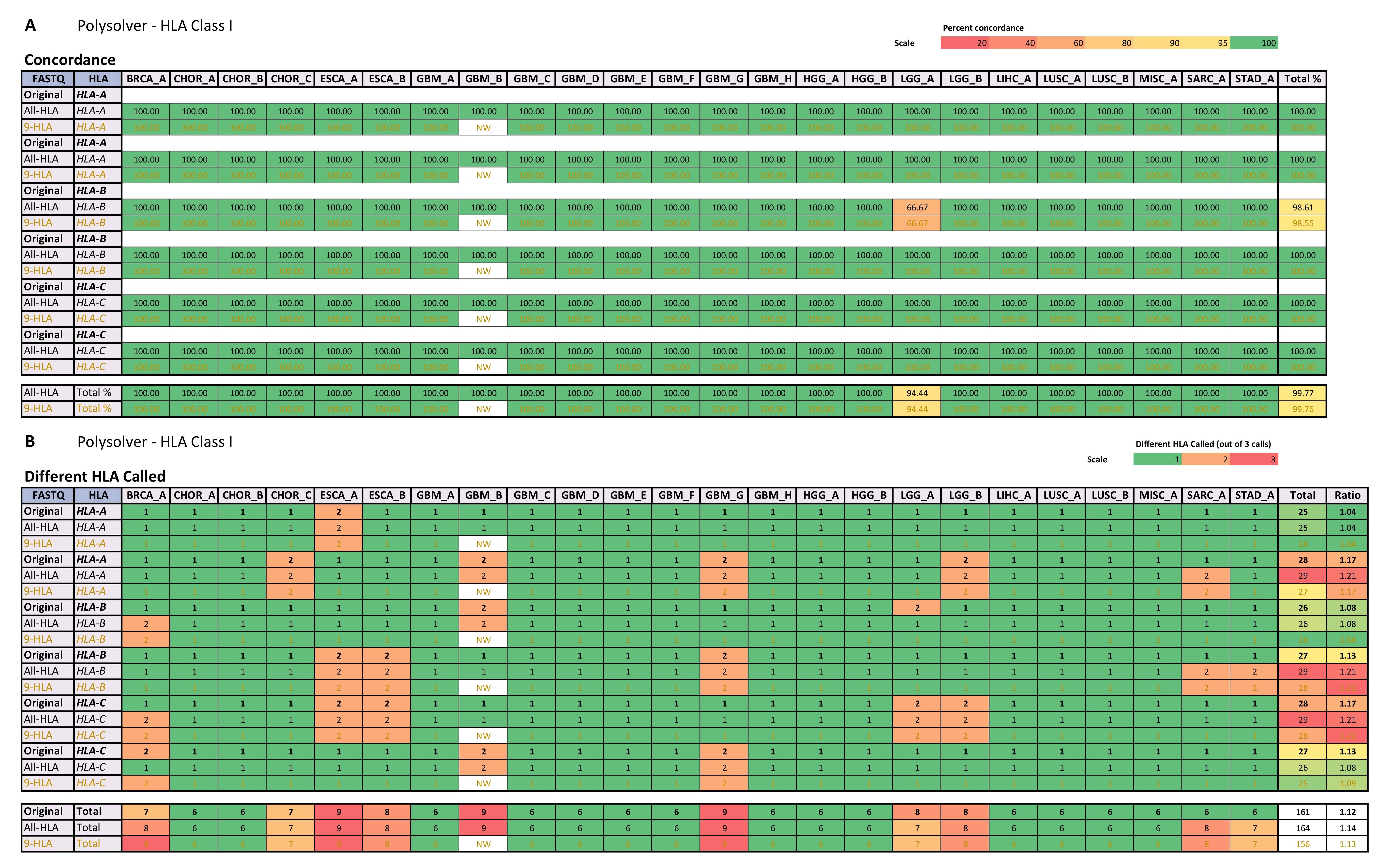

Supplement: Supplementary file 1 [file biology-14-01717-s001.zip › SupplementaryFigure-S7.jpg]

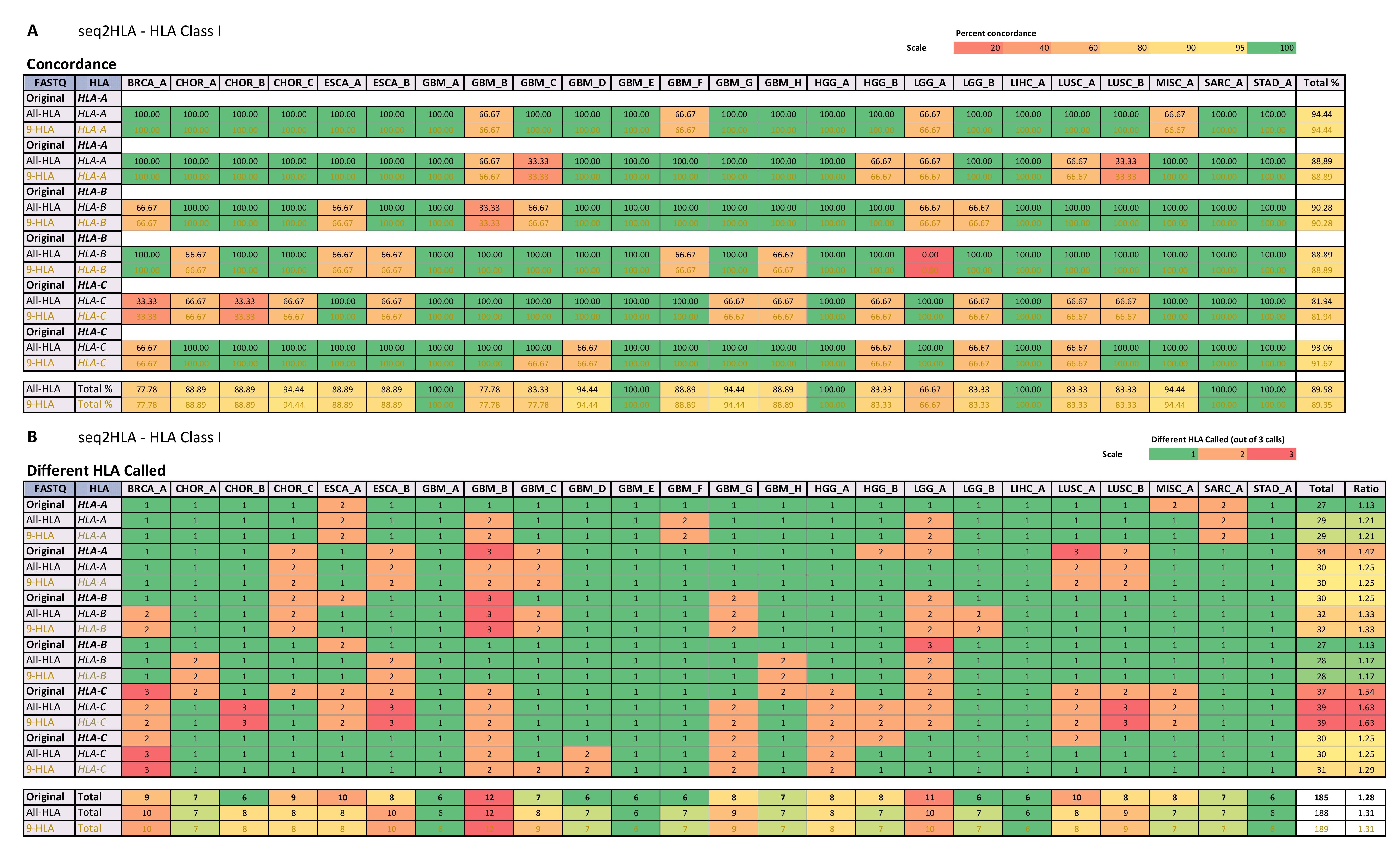

Supplement: Supplementary file 1 [file biology-14-01717-s001.zip › SupplementaryFigure-S8.jpg]

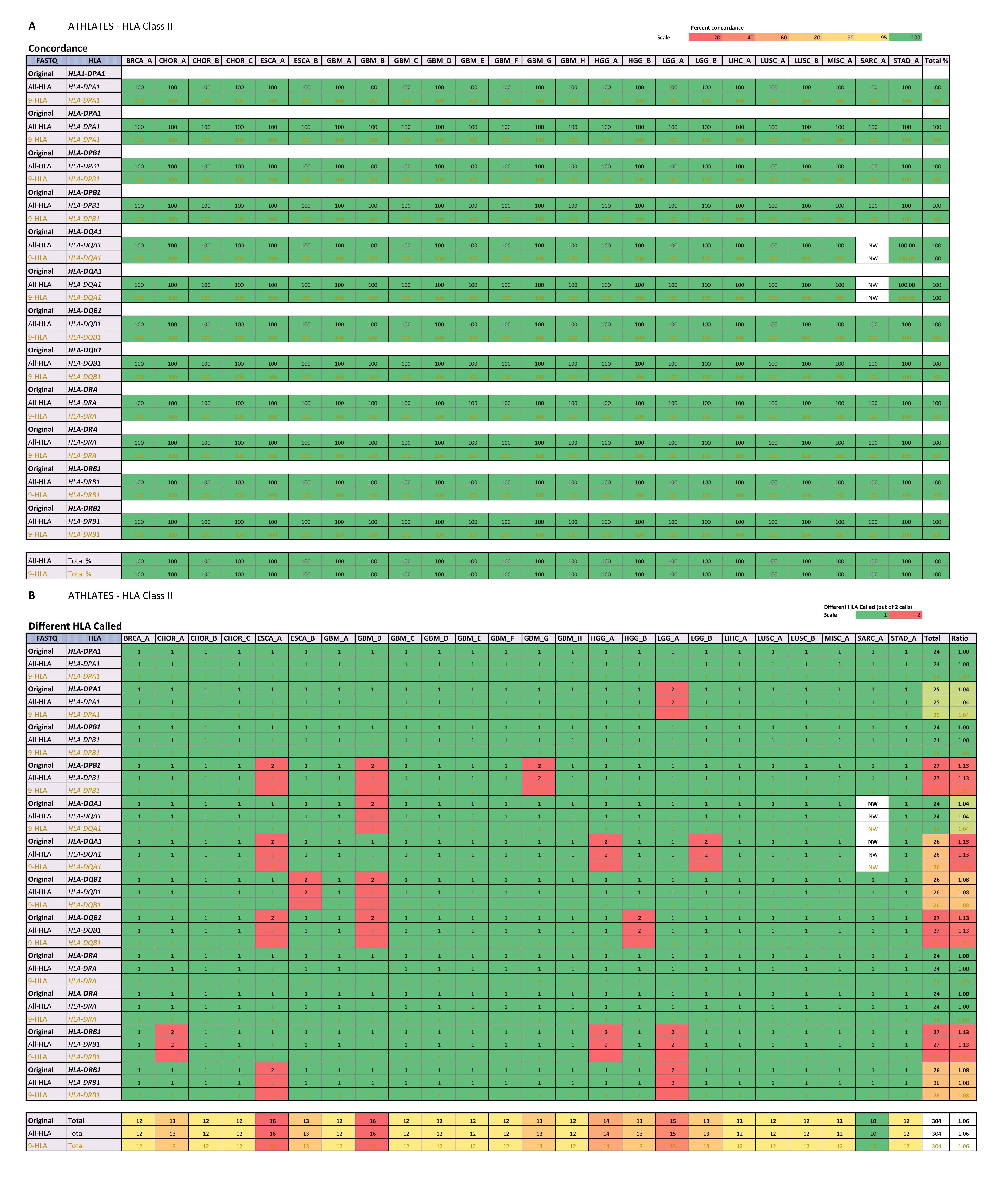

Supplement: Supplementary file 1 [file biology-14-01717-s001.zip › SupplementaryFigure-S9.jpg]
